# An efficient enantioselective approach to multifunctionalized γ-butyrolactone: concise synthesis of (+)-nephrosteranic acid†

**DOI:** 10.1039/d0ra04267f

**Published:** 2020-05-22

**Authors:** Anju Gehlawat, Ranjana Prakash, Satyendra Kumar Pandey

**Affiliations:** School of Chemistry and Biochemistry, Thapar Institute of Engineering and Technology Patiala-147 001 India skpandey@thapar.edu; Department of Chemistry, Institute of Science, Banaras Hindu University Varanasi-221 005 India skpandey.chem@bhu.ac.in

## Abstract

A short, efficient and novel approach for multifunctionalized γ-butyrolactone paraconic acids and its application to the total synthesis of (+)-nephrosteranic acid from readily available PMB (*R*)-glycidyl ether as a starting material are described. Key transformations include asymmetric Michael addition catalyzed by chiral diphenylprolinol silyl ether and stereoselective α-methylation.

## Abstract 2

A short, efficient and novel approach for multifunctionalized γ-butyrolactone paraconic acids and its application to the total synthesis of (+)-nephrosteranic acid from readily available PMB (*R*)-glycidyl ether as a starting material are described.

## Graphical abstract

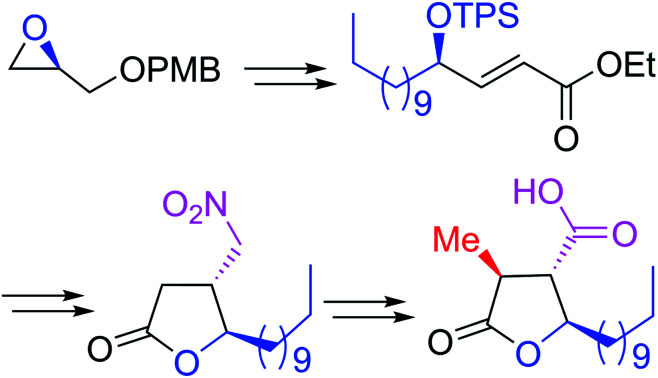

Bioactive natural products containing a multifunctionalized γ-butyrolactone moiety are found abundantly in nature.^1^ The paraconic acids (1–10) containing multifunctionalized γ-butyrolactone were isolated from various species of lichens, fungi, moss and cultures of *Penicillium* sp.^2^ These acids possess interesting biological activities such as antitumor, antibacterial, antibiotic, antifungal/antiviral and growth regulatory properties.^3^ The whiskey lactone 11 and cognac lactone 12 have great commercial interest because they are potential key components in the flavor of aged alcoholic beverages.^4^ Architecturally, the paraconic acid family comprises a variable length alkyl chain at the C5 position, a C4 carboxyl group and methyl or methylene substituents at the C3 position, which play an important role in the biological activities of the paraconic acids (Fig. 1).

**Fig. 1:**
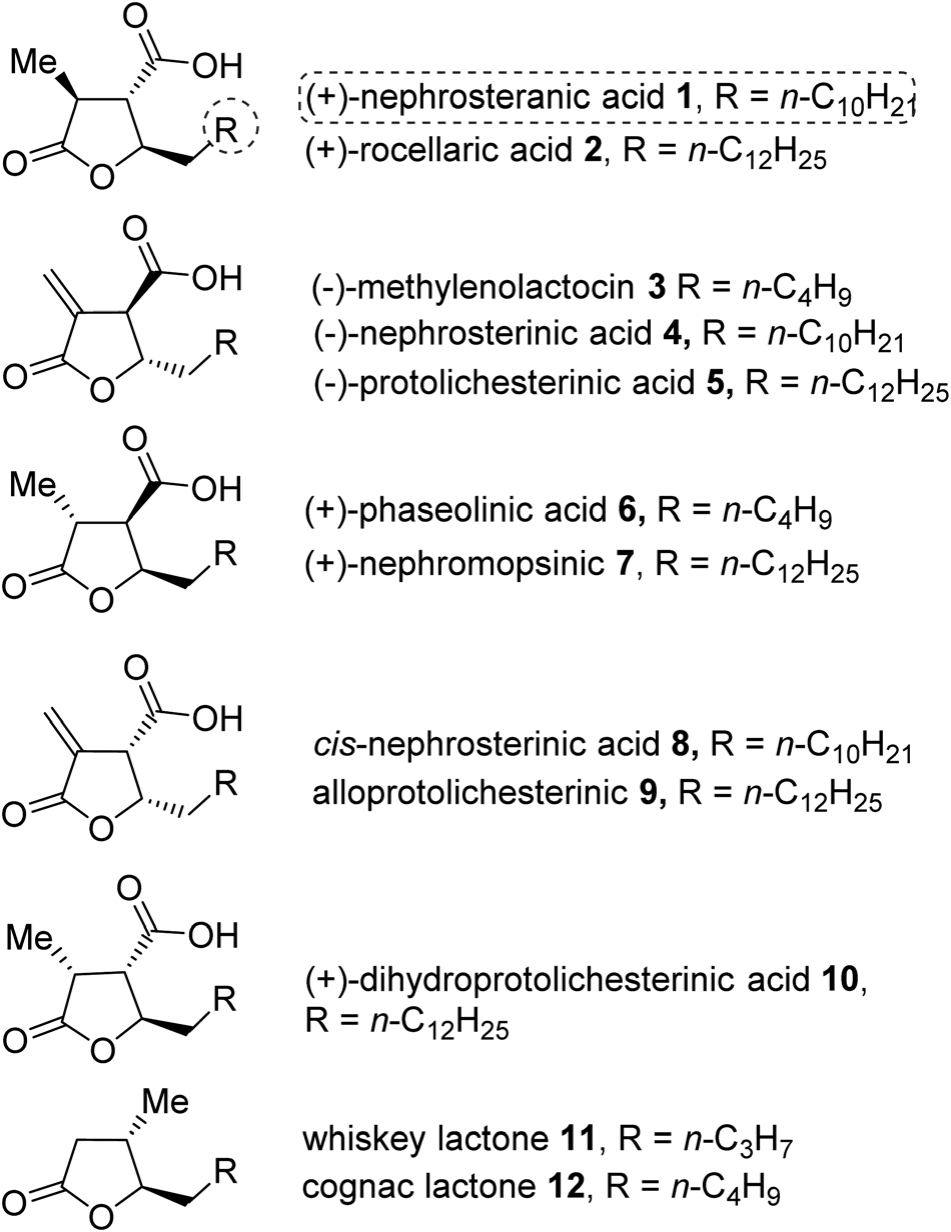
Representative structures of γ-butyrolactone based paraconic acids and lactones.

Intrigued by the unique structural features and biological activities of paraconic acids, hitherto, several total^5^ and formal^6^ synthesis of paraconic acids such as (+)-nephrosteranic acid are documented in literature. More recently, Appayee and co-workers disclosed an elegant approach for the stereodivergent synthesis of chiral paraconic acids *via* dynamic kinetic resolution of 3-acylsuccinimides.^7^ As part of our research program aimed at developing the asymmetric synthesis of bioactive natural molecules,^8^ we became attentive in developing a flexible and general approach for the synthesis of multifunctionalised γ-butyrolactone paraconic acids. Herein, we are reporting a short, efficient and novel general approach for the synthesis of paraconic acids and its application to the enantioselective synthesis of (+)-nephrosteranic acid 1 using organocatalyzed Michael addition reaction as key step.

Our general retrosynthetic route for asymmetric synthesis of γ-butyrolactone based paraconic acids and its application to enantioselective synthesis of (+)-nephrosteranic acid 1 was envisaged *via* the retrosynthetic approach as displayed Scheme 1. We envisioned that the γ-butyrolactone 13 could be used as a key intermediate from which paraconic acids 1–10 including (+)-nephrosteranic acid 1 would be synthesized *via* methylenation or stereoselective methylation at the C3 centre. The γ-butyrolactone 13 could be achieved from protected nitro-acid derivative 14*via* deprotection and *in situ* lactonization followed by Nef reaction. The nitro-acid derivative 14 in turn could be synthesized from (*R*)- or (*S*)-diphenylprolinol silyl ether catalyzed Michael addition of CH_3_NO_2_ to α,β-unsaturated aldehyde intermediate obtained from the controlled DIBAL-H reduction of olefinic ester derivative 15 followed by oxidation. The α,β-unsaturated ester 15 could be obtained from protected (*R*)- or (*S*)-glycidol 16 by treatment with suitable Grignard reagents, secondary alcohol protection, primary alcohol deprotection and oxidation followed by 2C-Wittig olefination reaction. The desired stereochemistry of paraconic acids 1–10 could be achieved by simply altering the (*R*)- and (*S*)-configuration of glycidyl ether and/or by using catalyst (*R*)- or (*S*)-diphenylprolinol silyl ether during Michael addition reaction. Thus, in principle, C3, C4 and C5 chiral centres in paraconic acids could be easily manipulated and accessed by this approach.

**Scheme 1:**
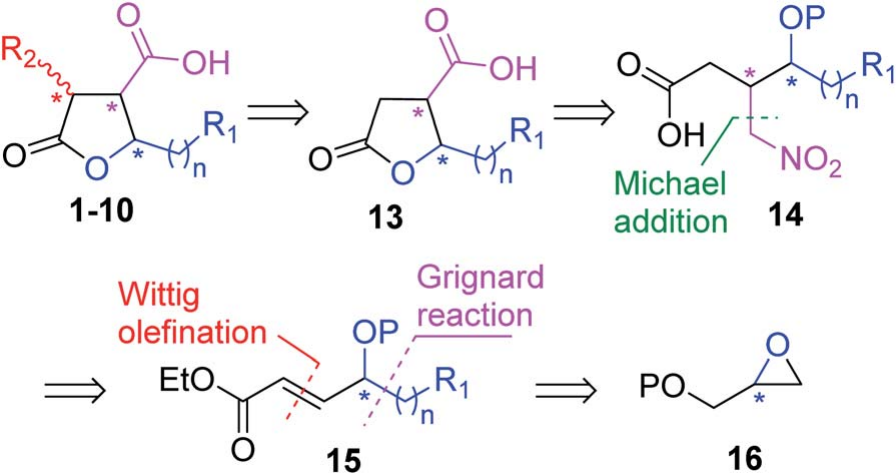
Retrosynthetic general approach of γ-butyrolactone based some paraconic acids and lactones.

As depicted in Scheme 2, the synthesis of (+)-nephrosteranic acid 1 as a representative target compound of paraconic acids was commenced from readily available PMB (*R*)-glycidyl ether 16a^9^ which was subjected to copper-catalyzed (CuI) regioselective ring opening with the Grignard reagent, derived from decyl bromide to furnish the alcohol derivative 17 in 85% yield. The alcohol derivative 17 on silyl protection with *tert*-butyldiphenylsilyl chloride (TBDPSCl) and imidazole with DMAP in catalytic amount afforded the silyl ether derivative 18 in 95% yield which on PMB ether cleavage using CAN (ceric ammonium nitrate) at 0 °C to rt furnished the terminal alcohol derivative 19 in 91% yield. The alcohol derivative 19 on oxidation under Swern conditions^10^ followed by treatment with (ethoxycarbonylmethylene)triphenylphosphorane in THF afforded the *trans*-olefinic ester derivative 20 in 92% yield. Our next goal was to carry out the synthesis of multifunctionalized γ-butyrolactone. Towards this end, *trans*-olefinic ester 20 on controlled reduction with DIBAL-H at −78 °C to α,β-unsaturated aldehyde intermediate and successive conjugate Michael addition^11^ of nitromethane in the presence of (*S*)-diphenylprolinol silyl ether (10 mol%) afforded the nitroaldehyde adduct which on subsequent oxidation with oxone^12^ furnished the nitro-acid derivative 21 in excellent yield.

**Scheme 2:**
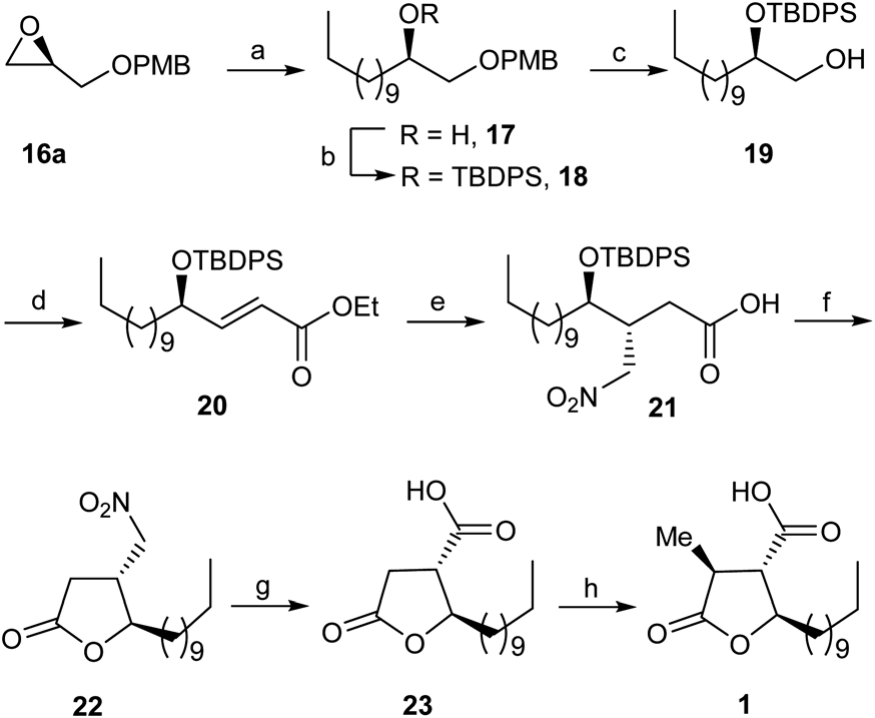
Reagents and conditions: (a) C_10_H_21_MgBr, CuI, dry THF, −30 °C, 6 h, 85%; (b) TBDPSCl, imidazole, cat. DMAP, CH_2_Cl_2_, 0 °C-rt, 8 h, 95%; (c) CAN, CH_3_CN : H_2_O (4 : 1, v/v), 0 °C-rt, 2 h, 91%; (d) (i) (COCl)_2_, DMSO, Et_3_N, CH_2_Cl_2_, −78 °C to −60 °C, 3 h; (ii) PPh_3_CHCOOEt, THF, rt, 20 h, 92% (over two steps); (e) (i) DIBAL-H, CH_2_Cl_2_, −78 °C, 1 h; (ii) (*S*)-diphenylprolinol silyl ether (10 mol%), CH_3_NO_2_, benzoic acid, MeOH, rt, 16 h; (iii) oxone, DMF, rt, 12 h, 84% (over 3 steps); (f) TBAF, dry THF, rt, 2 h, 95%; (g) NaNO_2_, acetic acid, DMSO, rt, 24 h, 94%; (h) NaHMDS, CH_3_I, dry THF, −78 °C, 3 h, 93%.

Further to demonstrate the stereochemistry during the conjugate Michael addition of nitromethane to α,β-unsaturated aldehyde intermediate we carried out the reaction with racemic catalyst (±)-diphenylprolinol silyl ether to get the nitro-aldehyde adduct which on subsequent oxidation with oxone afforded the *anti*-/*syn*-nitro acid diastereomers (dr, 1 : 1) in 83% combined yield. However, on the other hand, in presence of (*S*)-diphenylprolinol silyl ether catalyst the conjugate addition of nitromethane on α,β-unsaturated aldehyde intermediate obtained from 20 followed by oxidation with oxone furnished the *anti*-nitro acid derivative 21 as a single diastereomer^13^ in 84% yield.

The *anti*-nitro acid derivative 21 on TPS deprotection and concomitant cyclisation with TBAF (tetra-*n*-butylammonium fluoride) furnished the γ-butyrolactone derivative 22 in 95% yield. The nitro-γ-butyrolactone derivative 22 was subjected to treatment with sodium nitrite and acetic acid under Nef reaction conditions^14^ to afford the γ-butyrolactone acid derivative 23 in 94% yield. Finally, stereoselective methylation at α-position of acid derivative 23 was carried out with methyl iodide and NaHMDS in dry THF to furnish the target compound (+)-nephrosteranic acid 1 in 93% yield ([*α*]^25^_D_ + 27.18 (*c* 1.50, CHCl_3_), {lit.^5*s*^ [*α*]^27^_D_ + 27.2 (*c* 1.45, CHCl_3_)}. The spectral and physical properties of the (+)-nephrosteranic acid 1 were in full agreement with reported values.^5*a*^

## Conclusions

In summary, we have developed an efficient and enantioselective route to multifunctionalized γ-butyrolactone paraconic acids and its application to the synthesis of the (+)-nephrosteranic acid 1 from readily accessible PMB (*R*)-glycidyl ether as starting material. Pivotal reaction sequence comprises asymmetric Michael addition catalyzed by (*S*)-diphenylprolinol silyl ether and stereoselective α-methylation. The overall yield for the (+)-nephrosteranic acid 1 was 47%. The synthetic route presented has further potential for the stereochemical variations in all the positions of the ring and extension to other analogues.

## Conflicts of interest

There are no conflicts to declare.

## Supplementary Material

RA-010-D0RA04267F-s001
