# Supplementary material for: An efficient enantioselective approach to multifunctionalized γ-butyrolactone: concise synthesis of (+)-nephrosteranic acid
Source: RSC Adv. 2020 May 22;10(33):19655–8. doi: 10.1039/d0ra04267f (PMC9054058; doi:10.1039/d0ra04267f)
Supplement: RA-010-D0RA04267F-s001 [file RA-010-D0RA04267F-s001.pdf]

Electronic Supplementary Material (ESI) for RSC Advances.
This journal is © The Royal Society of Chemistry 2020

Electronic Supplementary Information

An Efficient Enantioselective Approach to Multifunctionalized γ-Butyrolactone: Concise Synthesis of (+)-Nephrosteranic Acid

Anju Gehlawat,[a] Ranjana Prakash[a] and Satyendra Kumar Pandey*[a,b]

[a]School of Chemistry and Biochemistry, Thapar Institute of Engineering and Technology, Patiala-147 001, India, E-mail: skpandey@thapar.edu

[b]Department of Chemistry, Institute of Science, Banaras Hindu University, Varanasi -221 005, India, Email: skpandey.chem@bhu.ac.in

Table of contents

1. General information and Experimental Section **S2-S7**
2. ^{1}H and ^{13}C spectra for compound **17** **S8-S9**
3. ^{1}H and ^{13}C spectra for compound **18** **S10-S11**
4. ^{1}H and ^{13}C spectra for compound **19** **S12-S13**
5. ^{1}H and ^{13}C spectra for compound **20** **S14-S15**
6. ^{1}H and ^{13}C spectra for *anti-*/*syn*-diastereomeric mixture of **21** **S16-S17**
7. ^{1}H and ^{13}C spectra for compound **21** **S18-S19**
8. ^{1}H and ^{13}C spectra for compound **22** **S20-S21**
9. ^{1}H and ^{13}C spectra for compound **23** **S22-S23**
10. ^{1}H and ^{13}C spectra for compound **1** **S24-S25**
11. ^{1}H and ^{13}C spectra for compound **1** (*known in literature, ref. cited*) **S26**

General methods

All reactions were carried out under argon or nitrogen in oven-dried glassware using standard glass syringes and septa. The solvents and chemicals were purchased from Merck and Sigma Aldrich chemical company. Solvents and reagents were purified and dried by standard methods prior to use. Progress of the reactions was monitored by TLC using precoated aluminium plates of Merck kieselgel 60 F254. Column chromatography was performed on silica gel (60-120 and 100-200 mesh) using a mixture of *n*-hexane and ethyl acetate. Optical rotations were measured on automatic polarimeter AA-65. IR spectra were recorded on Agilent resolution Pro 600 FT-IR spectrometer, fitted with a beam condensing ATR accessory. ^{1}H and ^{13}C NMR spectra were recorded in $CDCl_3$ (unless otherwise mentioned) on JEOL ECS operating at 400 and 100 MHz, respectively. Chemical shifts are reported in δ (ppm), referenced to TMS.

Experimental Section:

(*R*)-1-((4-methoxybenzyl)oxy)tridecan-2-ol, 17

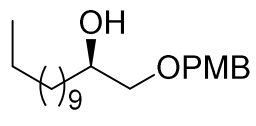

17

A solution of decylmagnesium bromide freshly prepared from Mg turnings (0.493 g, 20.58 mmol) and decylbromide (3.40 g, 15.43 mmol) in dry THF, was added dropwise to a stirred solution of (*R*)-PMB-glycidyl ether **15a** (2.0 g, 10.29 mmol) and CuI (195 mg, 1.029 mmol) in dry THF (20 mL) at -30 °C. The reaction mixture was stirred for 6 hours at the same temperature. After completion of reaction as monitored by TLC the reaction was quenched with saturated aq. NH_4Cl and extracted with ethyl acetate (3 x 20 mL), dried over anhydrous Na_2SO_4, concentrated *in vacuo* and purified by silica gel column chromatography to afford the (*R*)-alcohol derivative **17** (2.95 g, 85%) as white solid. R_f = 0.4 (hexane/EtOAc, 9.8:0.2, v/v); m.p. 186-187 ºC; $[\alpha]_D^{25}$ = - 2.78 (*c* = 1.0, $CHCl_3$); IR (CH_2Cl_2) ν: 3460, 2980, 1662, 1595, 1516, 1057, 937 cm^{-1}; ^{1}H NMR (400 MHz, $CDCl_3$) δ: 7.26 (d, *J* = 8.72 Hz, 2H), 6.89 (d, *J* = 8.72 Hz, 2H), 4.47 (s, 2H), 3.80 (s, 3H), 3.75-3.81 (m, 1H), 3.47 (dd, *J* = 9.64, 3.24 Hz, 1H), 3.28 (dd, *J* = 9.60, 8.24 Hz, 1H), 2.41 (brs, 1H), 1.33-1.47 (m, 2H), 1.25-1.32 (m, 18H), 0.87 (t, *J* = 6.4 Hz, 3H); ^{13}C NMR (100 MHz, $CDCl_3$) δ: 159.2, 130.0, 129.4, 113.8, 74.3, 72.9, 70.4, 55.2, 33.1, 31.9, 29.63, 29.59, 29.32, 25.5, 22.7, 14.1; HRMS (ESI), calcd for $C_{21}H_{36}O_3$ $[M+H]^+$: 337.2737, found 337.2741

(*R*)-*tert*-butyl((1-((4-methoxybenzyl)oxy)tridecan-2-yl)oxy)diphenylsilane, 18

To a stirred solution of alcohol derivative **17** (2.0 g, 5.94 mmol) in dry CH_2Cl_2 (30 mL) sequentially imidazole (606 mg, 8.91 mmol), *tert*-butylchlorodiphenylsilane (1.95 g, 7.12 mmol) and DMAP (108 mg, 0.89 mmole) were added at 0 ºC and further the reaction mixture was continued to stirred at room temperature for 8 hours. After completion of reaction as monitored by TLC the reaction was quenched with saturated aq. ammonium chloride solution. The aqueous layer was extracted with CH_2Cl_2 (3 x 20 mL). The combined organic phase was washed with brine, dried over anhydrous Na_2SO_4 and concentrated under reduced pressure. Purification of crude product by silica gel column chromatography (EtOAc/hexane, 0.1:9.9) furnished the silyl ether derivative **18** (3.24 g) in 95% yield as colorless oil. R_f = 0.5 (EtOAc/hexane 0.1:9.9); $[\alpha]_D^{25}$ = +8.91 (*c* = 1.0, $CHCl_3$); ^{1}H NMR (400 MHz, $CDCl_3$) δ: 7.66 (tt, *J* = 5.96, 1.34 Hz, 4H), 7.31-7.41 (m, 6H), 7.08 (d, *J* = 8.72 Hz, 2H), 6.81 (d, *J* = 8.72 Hz, 2H), 4.25 (q, *J* = 18.80, 11.44 Hz, 2H), 3.82-3.86 (m, 1H), 3.79 (s, 3H), 3.30-3.37 (m, 2H), 1.11-1.50 (m, 2H), 1.19-1.31(m, 18H), 1.03 (s, 9H), 0.88 (t, *J* = 7.12 Hz, 3H); ^{13}C NMR (100 MHz, $CDCl_3$) δ: 158.9, 136.0, 135.9, 134.6, 134.1, 130.5, 129.5, 129.3, 129.2, 127.3, 127.2, 113.5, 73.6, 72.6, 72.2, 55.2, 34.3, 31.9, 29.7, 29.6, 29.5, 29.4, 27.0, 24.7, 22.7, 19.4, 14.1; HRMS (ESI), calcd for $C_{37}H_{54}O_3Si$ $[M+Na]^+$: 597.3734, found 597.3737.

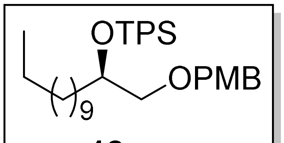

18

(*R*)-2-((*tert*-butyldiphenylsilyl)oxy)tridecan-1-ol, 19

To a stirred solution of **18** (2.50 g, 4.34 mmol) in acetonitrile: water (4:1 = v/v, 30 mL) at 0 ºC CAN (5.76 g, 10.52 mmol) was added and the reaction mixture was stirred at room temperature for 2.0 hours. After completion of reaction as monitored by TLC, the reaction was quenched with brine (5 mL) solution and the aqueous layer was extracted with ethyl acetate (3 x 15 mL). The combined organic portion was dried over anhydrous Na_2SO_4 and concentrated in *vacuo*. The residue was purified by silica gel column chromatography (EtOAc/hexane 0.5:9.5) to afford the primary alcohol derivative **19** (1.79 g) in 91% yield as a white solid. R_f = 0.5 (EtOAc/hexane 0.5:9.5); m.p. 147-148 ºC; $[\alpha]_D^{25}$ = -29.21 (*c* = 1.0, $CHCl_3$); IR (CH_2Cl_2) *v*: 3329, 2755, 1660, 1589, 1581, 1509, 1161, 910 cm^{-1}; ^{1}H NMR (400 MHz, $CDCl_3$) δ: 7.68 (dt, *J* = 8.04, 1.64 Hz, 4H), 7.38-7.45 (m, 6H), 3.73-3.79 (m, 1H), 3.45-3.57 (m, 2H), 1.84 (brs, 1H), 1.39-1.49 (m, 2H), 1.11-1.33 (m, 18H), 1.07 (s, 9H), 0.88 (t, *J* = 7.12 Hz, 3H); ^{13}C NMR (100 MHz, $CDCl_3$) δ: 135.9, 135.7, 133.9, 133.8, 129.8, 129.7, 127.7, 127.6, 74.1, 65.9, 33.5, 31.9, 29.6, 29.5, 29.4, 29.3, 27.0, 25.1, 22.7, 19.3, 14.1; HRMS (ESI), calcd for $C_{29}H_{46}O_2Si$ $[M+Na]^+$: 477.3159, found: 477.3161.

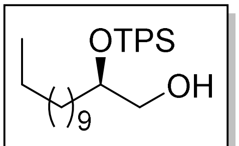

19

ethyl (*R*,*E*)-4-((*tert*-butyldiphenylsilyl)oxy)pentadec-2-enoate, 20

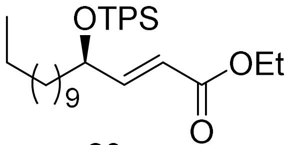

20

To a stirred solution of oxalyl chloride (628 mg, 0.427 mL, 4.95 mmol) in dry CH_2Cl_2 (2 mL) at -78 ºC was added DMSO (798 mg, 0.725 mL, 10.24 mmol) in dry CH_2Cl_2 (2 mL) dropwise and stirred the reaction mixture for 30 min and then a solution of silyl protected alcohol derivative **19** (1.50 g, 1.52 mmol) in dry CH_2Cl_2 (10 mL) was added dropwise over 15 min at the same temperature. The reaction mixture was stirred for 30 min at -78 °C and 1 hours at -60 °C and then Et_3N (1.46 g, 2.02 mL, 14.54 mmol) was added dropwise at the same temperature and stirred for an additional 1 hour. The reaction mixture was allowed to warm to room temperature and diluted with water and CH_2Cl_2. The organic layer was separated and the aqueous layer was extracted with CH_2Cl_2 (3 x 15 mL) and the combined organic layer was washed with brine, dried over anhydrous Na_2SO_4, and concentrated in vacuo to give the crude aldehyde, which was used in the next step after filter column purification.

To a solution of the above aldehyde in THF (10 mL) was added to a solution of (ethoxycarbonylmethylene)triphenylphosphorane (1.73 g, 4.95 mmol) in THF (20 mL). The reaction mixture was continuously stirred for 20 h at room temperature. After completion of reaction as monitored by TLC, it was then concentrated under reduced pressure and purified by silica gel column chromatography using EtOAc/hexane (1:9, v/v) as eluent to furnish the corresponding *α*, *β*- unsaturated ester derivative **20** (1.58 g, 92% over two steps) as a colorless thick syrupy liquid. R_f = 0.35 (EtOAc/hexane 1:9); $[\alpha]_D^{25}$ = +7.29 (*c* = 1.0, $CHCl_3$); IR (CH_2Cl_2) *v*: 2952, 2861, 1728, 1655, 1356, 1187 cm^{-1}; ^1H NMR (400 MHz, $CDCl_3$) *δ*: 7.68 (dd, *J* = 8.24, 1.36 Hz, 2H), 7.61 (dd, *J* = 7.80, 1.36 Hz, 2H), 7.35-7.45 (m, 6H), 6.86 (dd, *J* = 15.56, 5.04 Hz, 1H), 5.90 (dd, *J* = 15.56, 1.36 Hz, 1H), 4.34 (q, *J* = 6.44 Hz, 1H), 4.18 (dq, *J* = 14.20, 6.88, 2.33 Hz, 2H), 1.35-1.49 (m, 2H), 1.29 (t, *J* = 7.32, 3H), 1.11-1.29 (m, 18H), 1.08 (s, 9H), 0.88 (t, *J* = 6.88, 3H); ^{13}C NMR (100 MHz, $CDCl_3$) *δ*:166.7, 150.3, 135.8, 134.0, 133.5, 129.7, 129.6, 127.6, 120.0, 72.4, 60.3, 36.7, 31.9, 29.7, 29.6, 29.5, 29.4, 27.0, 26.9, 23.9, 22.7, 19.3, 14.2, 14.1; HRMS (ESI), calcd for $C_{33}H_{50}O_3Si$ $[M+H]^+$: 523.3602; found: 523.3599.

(3*R*,4*R*)-4-((*tert*-butyldiphenylsilyl)oxy)-3-(nitromethyl)pentadecanoic acid, 21

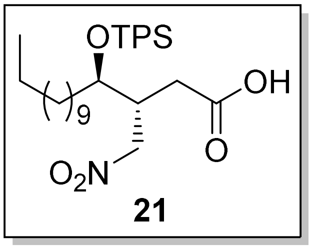

21

To a stirred solution of *α*, *β*-unsaturated ester derivative **20** (1.0 g, 1.92 mmol) in dry CH_2Cl_2 (15 mL) was added DIBAL-H (2.30 mL, 2.30 mmol, 1 M in hexane) under inert atmosphere at -78 °C. After stirring the reaction mixture for 1 hour at same temperature the reaction was quenched with saturated aqueous solution of sodium potassium tartrate and stirred for additional 30 min. The two phases were separated and aqueous phase was extracted with CH_2Cl_2 (3 x 15 mL). The combined organic layers extract was dried over anhydrous Na_2SO_4 and concentrated *in vacuo* to give *α*, *β*-unsaturated aldehyde which was used as such for the next step after filtration column using Celite.

To the above *α*, *β*-unsaturated aldehyde intermediate in dry methanol was added nitromethane (0.31 mL, 5.76 mmol), (*S*)-diphenyltrimethylsiloxymethylpyrrolidine (62 mg, 0.19 mmol) and benzoic acid (23 mg, 0.19 mmol) sequentially at room temperature. The reaction mixture was stirred for 16 h at room temperature. After completion of reaction as monitored by TLC the reaction was quenched with saturated aqueous $NaHCO_3$ solution. The organic layer was extracted with EtOAc (3 x 10 mL), dried over anhydrous Na_2SO_4 and concentrated in *vacuo* to furnish nitro-aldehyde intermediate which was used directly for the next step without further purification.

To a stirred solution of above nitro-aldehyde intermediate in DMF (10 mL) solvent oxone (2.30 g, 7.68 mmol) was added and the reaction mixture was stirred at room temperature for 12 hours. After completion of reaction as monitored by TLC, the reaction mixture was diluted with water and extracted with EtOAc (3 x 15 mL). The combined organic extract was washed with brine, dried over anhydrous Na_2SO_4, concentrated *in vacuo* to get the crude acid. Purification of crude product by silica gel column chromatography (EtOAc/hexane 2:8, v/v) furnished the nitro-acid derivative **21** (870 mg, 84%, over three steps) as colorless solid. R_f = 0.4 (EtOAc/hexane, 1:4); m.p. 161-162 ºC; $[\alpha]_D^{25}$ = + 16.52 (*c* = 1.0, $CHCl_3$); IR (CH_2Cl_2) *v*: 3152, 2831, 1728, 1527, 1362 cm^{-1}; ^{1}H NMR (400 MHz, $CDCl_3$) *δ*: *δ*: 7.65 (t, *J* = 1.36 Hz, 2H), 7.63 (t, *J* = 1.40 Hz, 2H), 7.46-7.36 (m, 6H), 4.62 (dd, *J* = 13.28, 7.32 Hz, 1H), 4.49 (dd, *J* = 12.84, 6.44 Hz, 1H), 3.72 (dt, *J* = 5.48, 2.28 Hz, 1H), 2.83-2.92 (m, 1H), 2.67 (dd, *J* = 16.96, 5.04 Hz, 1H), 2.47 (dd, *J* = 16.92, 8.24 Hz, 1H), 1.11-1.45 (m, 20H), 1.04 (s, 9H), 0.88 (t, *J* = 6.4, 3H); ^{13}C NMR (100 MHz, $CDCl_3$) *δ*: 177.4, 135.8, 135.7, 134.8, 133.7, 132.4, 130.1, 129.7, 127.9, 127.7, 127.5, 76.4, 72.8, 38.1, 33.8, 31.9, 31.0, 29.6, 29.5, 29.4, 29.3, 29.2, 29.0, 27.0, 25.3, 22.7, 19.4, 14.1; HRMS (ESI), calcd for $C_{32}H_{49}NO_5Si$ $[M+H]^+$: 556.3453, found: 556.3455.

(4*R*,5*R*)-4-(nitromethyl)-5-undecyldihydrofuran-2(3H)-one, 22

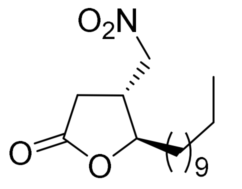

22

To a stirred solution of compound **21** (600 mg, 1.07 mmol) in dry THF (15 mL) TBAF (1.29 mL, 1.0 M in THF, 1.29 mmol) was added dropwise *via* syringe and the reaction mixture was stirred at room temperature for 2 hours. After completion of reaction as monitored by TLC the reaction was quenched with saturated aqueous NH_4Cl solution and aqueous phase was extracted with ethyl acetate (3 x 10 mL). The combined organic fractions were dried over anhydrous Na_2SO_4 and concentrated in *vacuo* to get the crude product. The residue was purified by silica gel column chromatography (EtOAc/hexane, 3:2 v/v) to afford the nitro γ-butyrolactone derivative **22** (315 mg, 95%) as a colorless solid. R_f = 0.4 (EtOAc/hexane 1.5:8.5); m.p. 137-138 °C; $[\alpha]_D^{25}$ = +28.26 (*c* = 1.0, $CHCl_3$); IR (CH_2Cl_2) *v*: 1768, 1534, 1514, 1056, 937 cm^{-1}; ^1H NMR (400 MHz, $CDCl_3$) *δ*: 4.53 (dd, *J* = 13.28, 6.88 Hz, 1H), 4.45 (dd, *J* = 13.28, 7.44 Hz, 1H), 4.28 (q, *J* = 12.84, 5.96 Hz, 1H), 2.90 (dd, *J* = 17.88, 9.16 Hz, 2H), 2.43 (dd, *J* = 17.88, 6.44 Hz, 1H), 1.72-1.66 (m, 2H), 1.26-1.45 (m, 18 H), 0.88 (t, *J* = 6.44, 3H); ^{13}C NMR (100 MHz, $CDCl_3$) *δ*: 174.1, 82.1, 76.4, 38.7, 34.7, 32.5, 31.9, 29.6, 29.5, 29.3, 29.3, 29.2, 25.2, 22.7, 14.1; HRMS (ESI), calcd for $C_{16}H_{29}NO_4$ $[M+H]^+$: 300.2169, found 300.2159

(2*R*,3*S*)-5-oxo-2-undecyltetrahydrofuran-3-carboxylic acid, 23

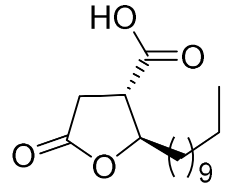

23

To a stirred solution of nitro γ-butyrolactone derivative **22** (150 mg, 0.40 mmol) in dimethyl sulfoxide (5 mL) sodium nitrite (83 mg, 1.20 mmol) and acetic acid (0.22 mL, 4.00 mmol) were added and the reaction mixture was stirred at room temperature for 24 hours. After completion of reaction as monitored by TLC the reaction mixture was diluted with water, acidified with 10% aqueous solution of HCl (2 mL), extracted with ether (3 x 10 mL), dried over anhydrous Na_2SO_4, concentrated *in vacuo*, and purified by silica gel column chromatography (EtOAc/hexane, 1:1 v/v) as eluent to furnish the γ-butyrolactone acid derivative **23** (134 mg, 94%) as a white solid. R_f = 0.4 (EtOAc/hexane 7:3); m.p. 117-118 °C; $[\alpha]_D^{25}$ = +45.11 (*c* = 0.25, $CHCl_3$) { lit.[SI-1] $[\alpha]_D^{25}$ +44.8 (*c* = 0.25, $CHCl_3$)}; ^1H NMR (400 MHz, $CDCl_3$) *δ*: 4.60-4.65 (m, 1H), 3.06-3.13 (m, 1H), 2.94 (dd, *J* = 17.88, 8.24 Hz, 1H), 2.82 (dd, *J* = 17.88, 9.64 Hz, 1H), 1.69-1.89 (m, 2H), 1.18-1.59 (m, 18 H), 0.88 (t, *J* = 6.88, 3H); ^{13}C NMR (100 MHz, $CDCl_3$) *δ*: 175.4, 174.4, 81.8, 45.4, 35.4, 31.9, 29.6, 29.5, 29.4, 29.3, 29.2, 25.2, 22.7, 14.1; HRMS (ESI), calcd for $C_{16}H_{28}O_4$ $[M+H]^+$: 285.2060, found 285.2064.

(+)-Nephrosteranic acid, 1

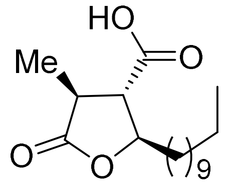

1

To a stirred solution of γ-butyrolactone acid derivative **23** (60 mg, 0.21 mmol) in dry THF (2 mL) was added NaHMDS (0.46 mL, 1.0 M solution in THF, 0.46 mmol) at -78 °C in drop wise fashion and stirred the reaction mixture for 1 hour. Further, MeI (281 mg, 0.13 mL, 2.00 mmol) was added and the reaction was allowed to stirred at -78 °C for additional 2 hours. After completion of reaction the reaction mixture as monitored by TLC the mixture was allowed to warm up to -20 ºC. The HCl (2N, 1.0 mL) was added to the reaction mixture and extracted with EtOAc (3 x 5 mL). The combined organic phases were dried over anhydrous Na_2SO_4 and concentrated under reduced pressure. The residue obtained was purified by preparative TLC (EtOAc/hexane, 1:1 v/v) to furnish the (+)-nephrosteranic acid **1** (58 mg, 93%) as white solid. R_f = 0.4 (EtOAc/hexane, 1:1); m.p. 96-97 ºC; $[\alpha]_D^{25}$ +27.18 (*c* 1.50, $CHCl_3$), {lit. [SI-1] $[\alpha]_D^{25}$ +27.2 (*c* 1.45, $CHCl_3$), lit. [SI-2] $[\alpha]_D^{25}$ +26.9 (*c* 0.14, $CHCl_3$)}; ^{1}H NMR (400 MHz, $CDCl_3$) δ: 4.48 (dt, *J* = 8.72, 3.68 Hz, 1H), 2.95-3.04 (m, 1H), 2.71 (dd, *J* = 11.48, 9.64 Hz, 1H), 1.66-1.87 (m, 2H), 1.37 (d *J* = 7.32 Hz, 3 H), 1.02-1.61 (m, 18H), 0.88 (t, *J* = 6.40, 3H); ^{13}C NMR (100 MHz, $CDCl_3$) δ: 176.7, 175.7, 79.4, 53.9, 39.8, 34.9, 31.9, 29.7, 29.6, 29.5, 29.4, 29.3, 29.2, 25.3, 22.7, 14.5, 14.1; HRMS (ESI), calcd for $C_{17}H_{30}O_4$ $[M+H]^+$: 299.2217; found: 299.2219.

References:

1. M. T. Barros, C. D. Maycock and M. R. Venture, *Org. Lett.*, **2003**, *5*, 4097-4099.
2. J. L. Nallasivam, R. A. Fernandes, *Org. Biomol. Chem.*, **2017**, *15*, 708-716.

^{1}H NMR (400 MHz, $CDCl_3$/TMS)

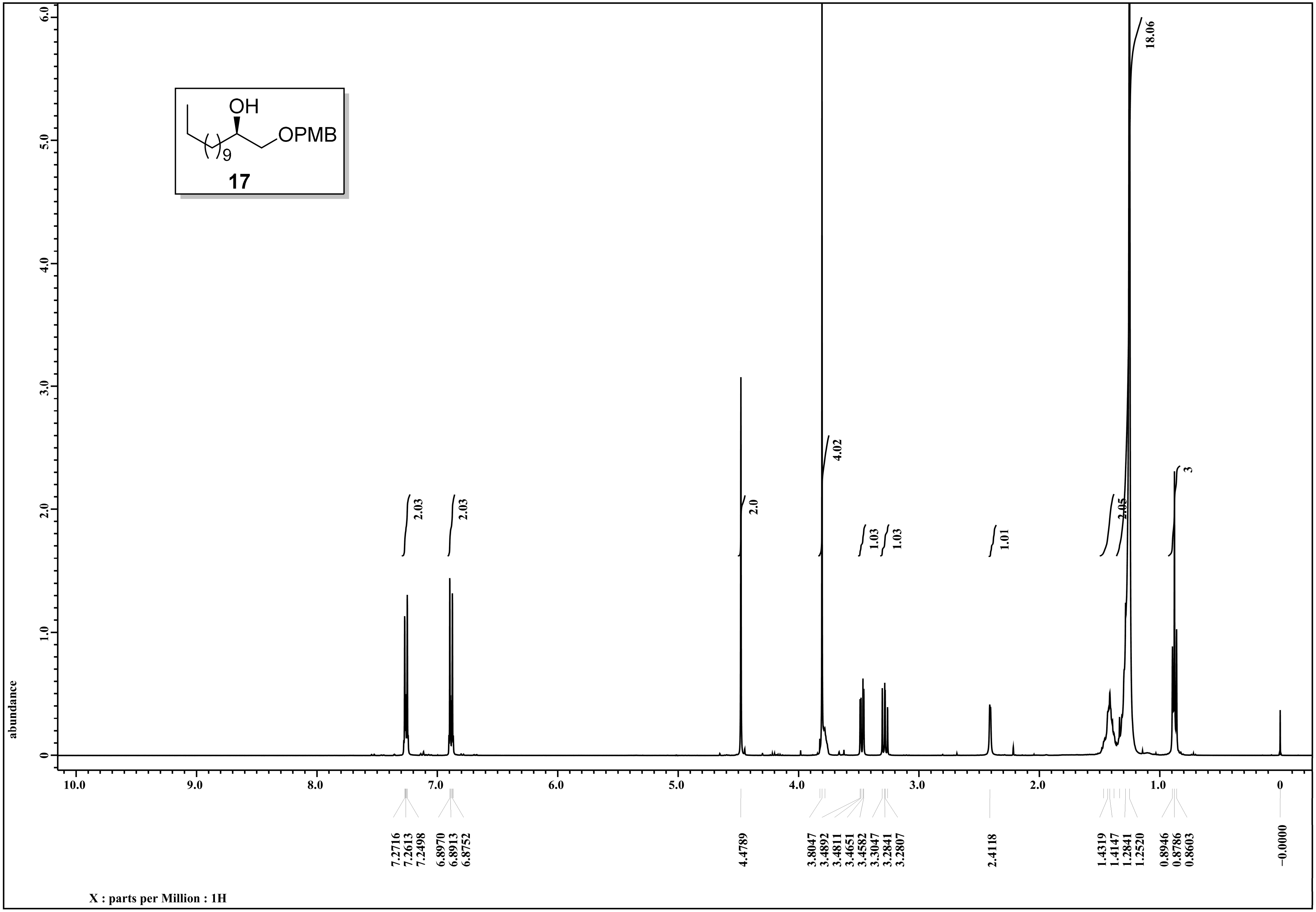

^{13}C NMR (100 MHz, $CDCl_3$/TMS)

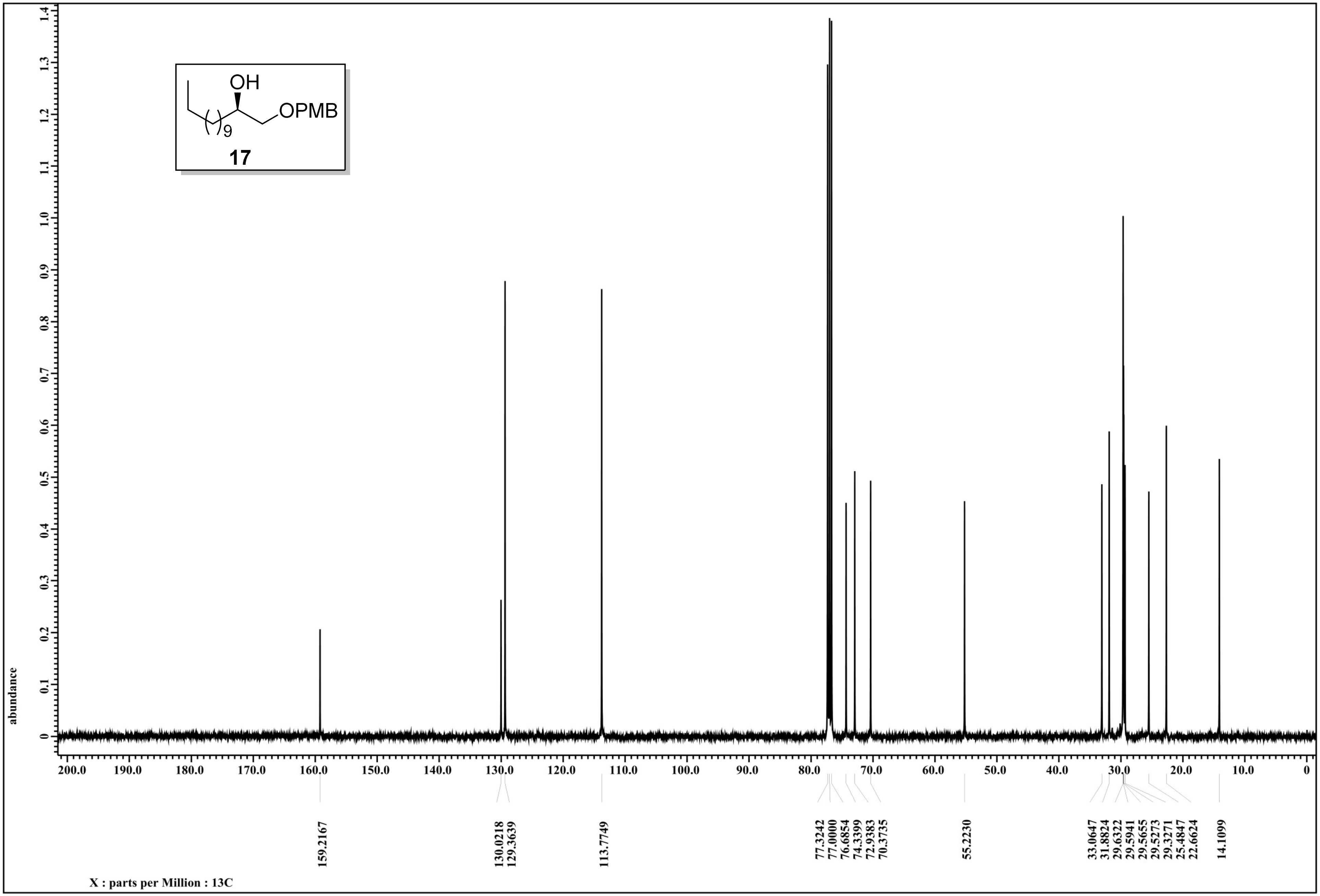

^{1}H NMR (400 MHz, $CDCl_3$/TMS)

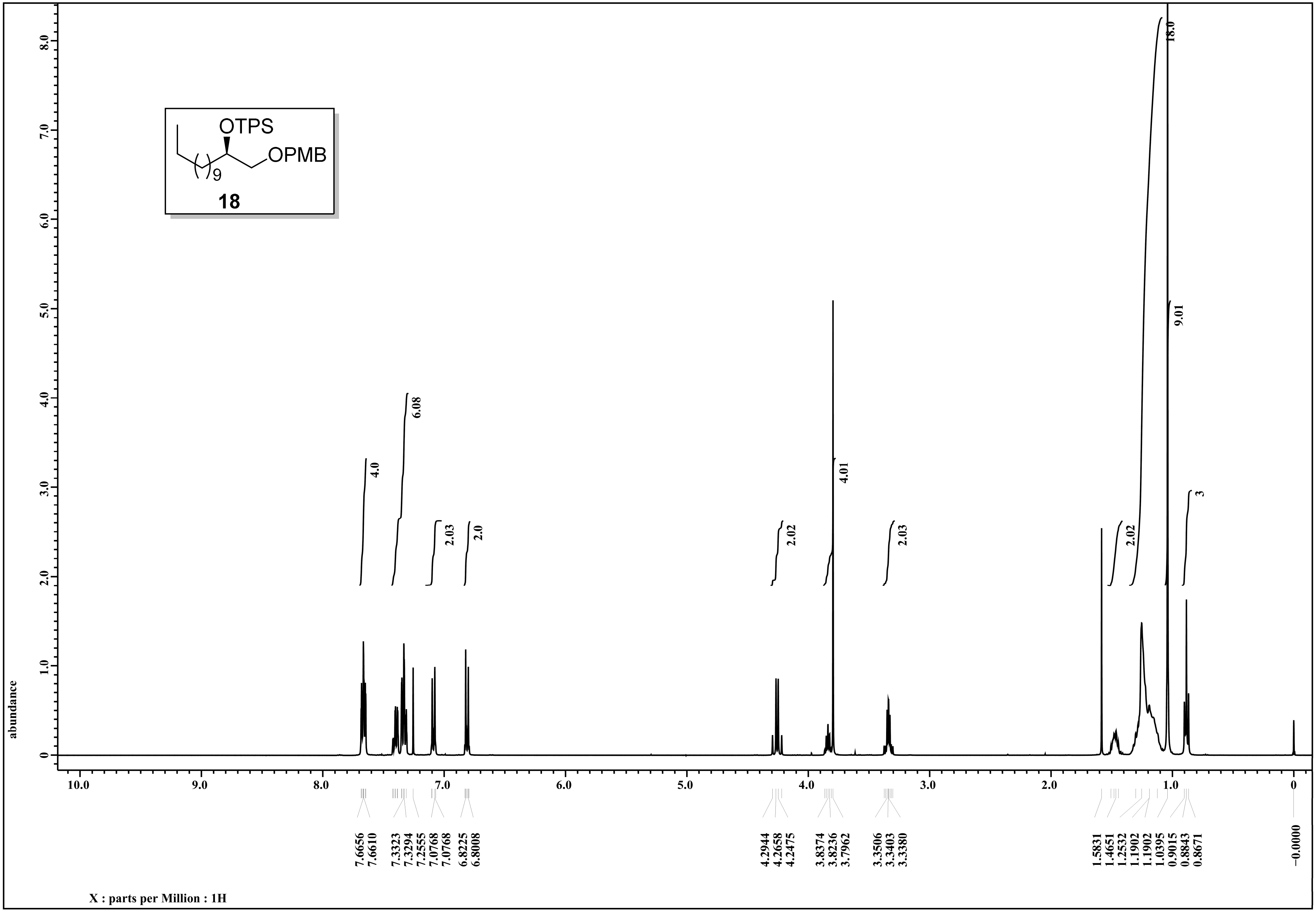

^{13}C NMR (100 MHz, $CDCl_3$/TMS)

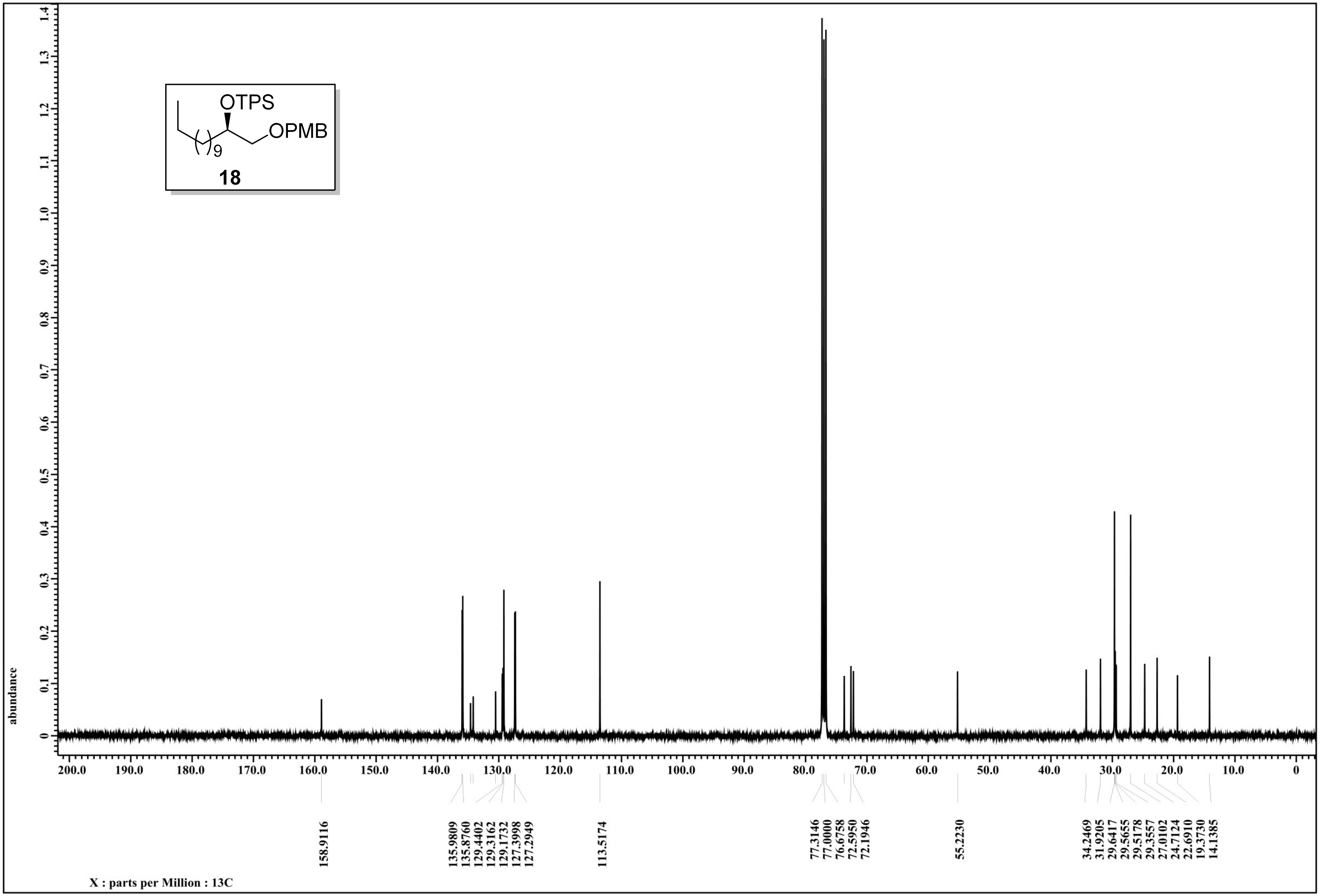

^{1}H NMR (400 MHz, $CDCl_3$/TMS

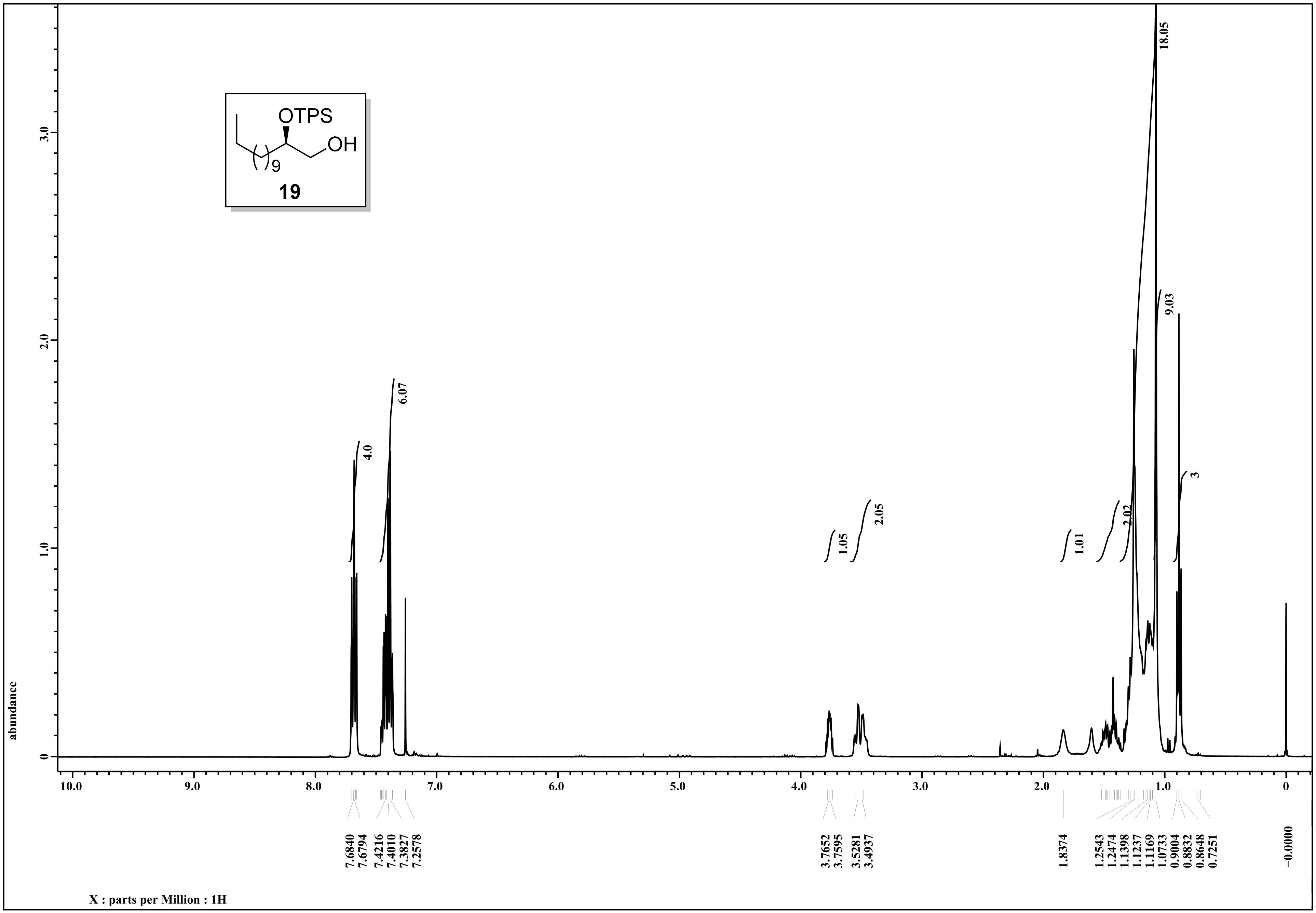

^{13}C NMR (100 MHz, $CDCl_3$/TMS)

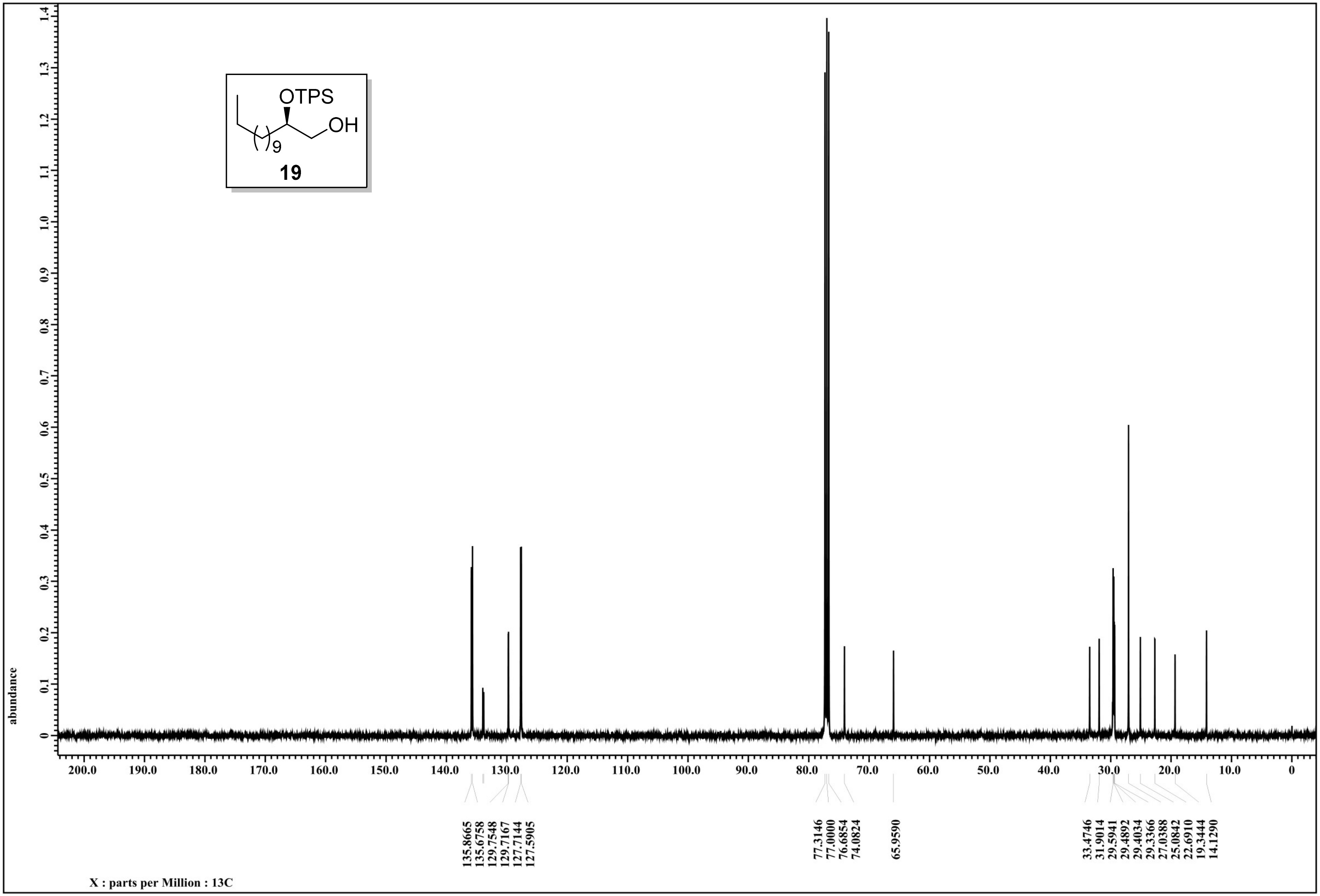

^{1}H NMR (400 MHz, $CDCl_3$/TMS)

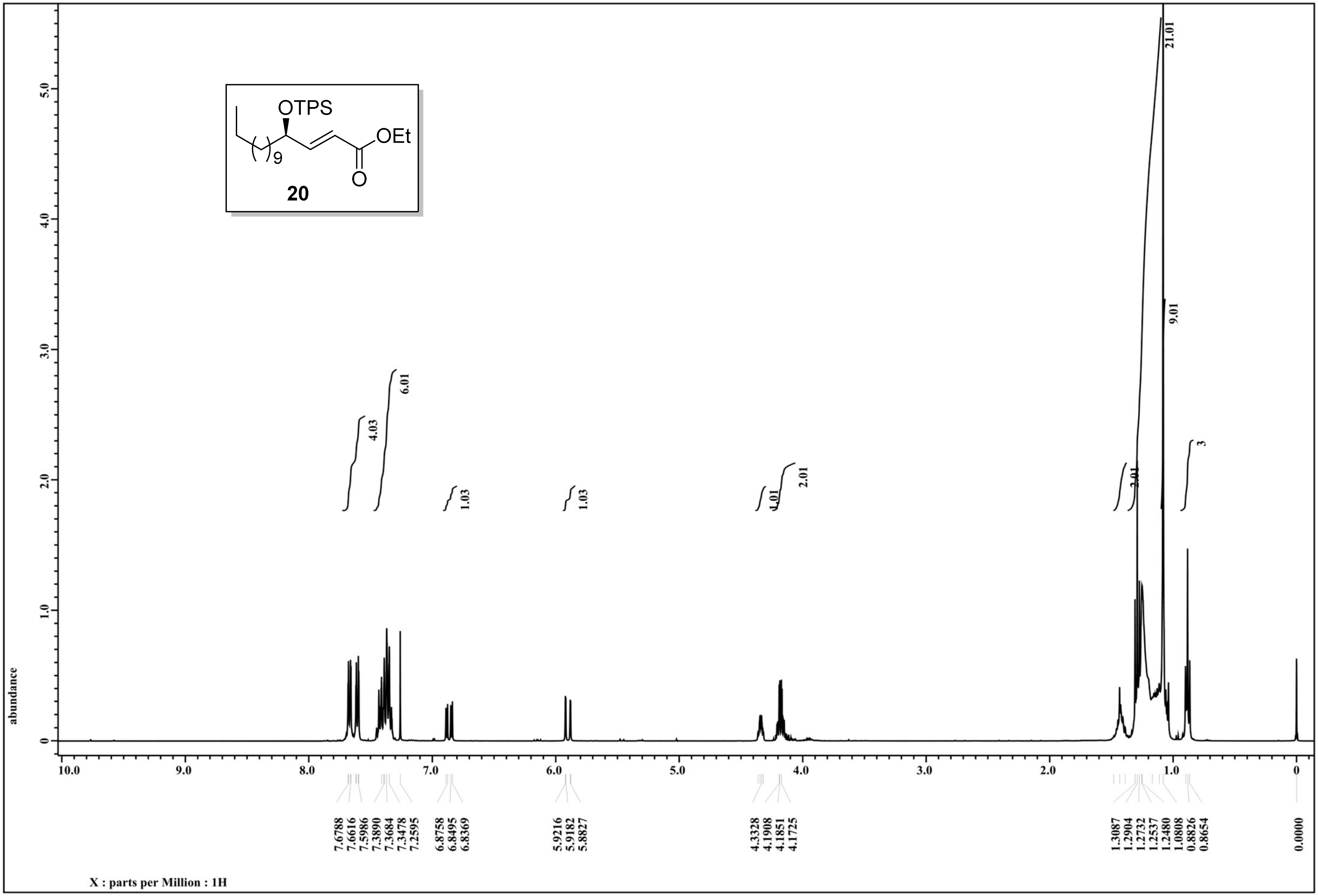

^{13}C NMR (100 MHz, $CDCl_3$/TMS)

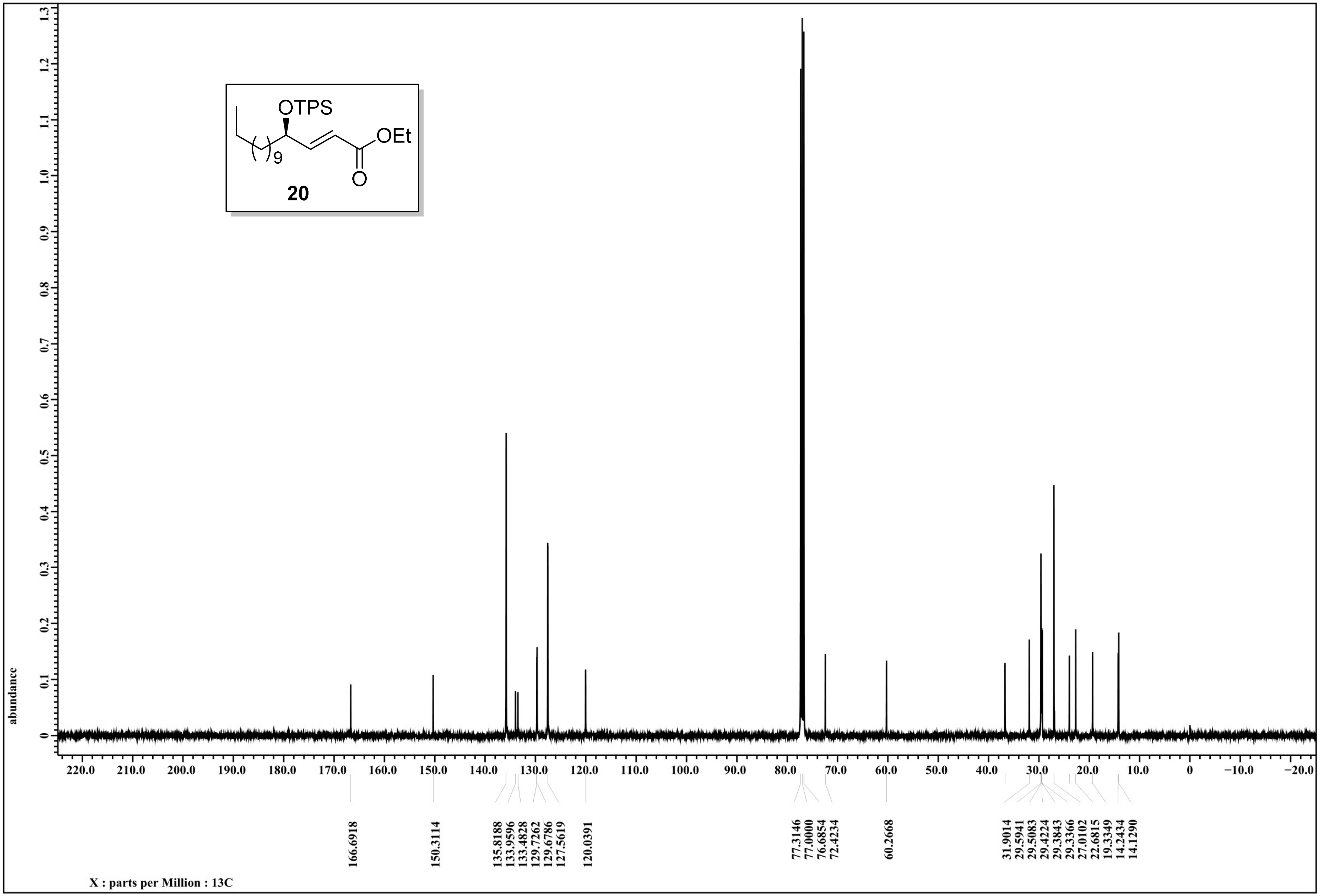

^{1}H NMR (400 MHz, $CDCl_3$/TMS)

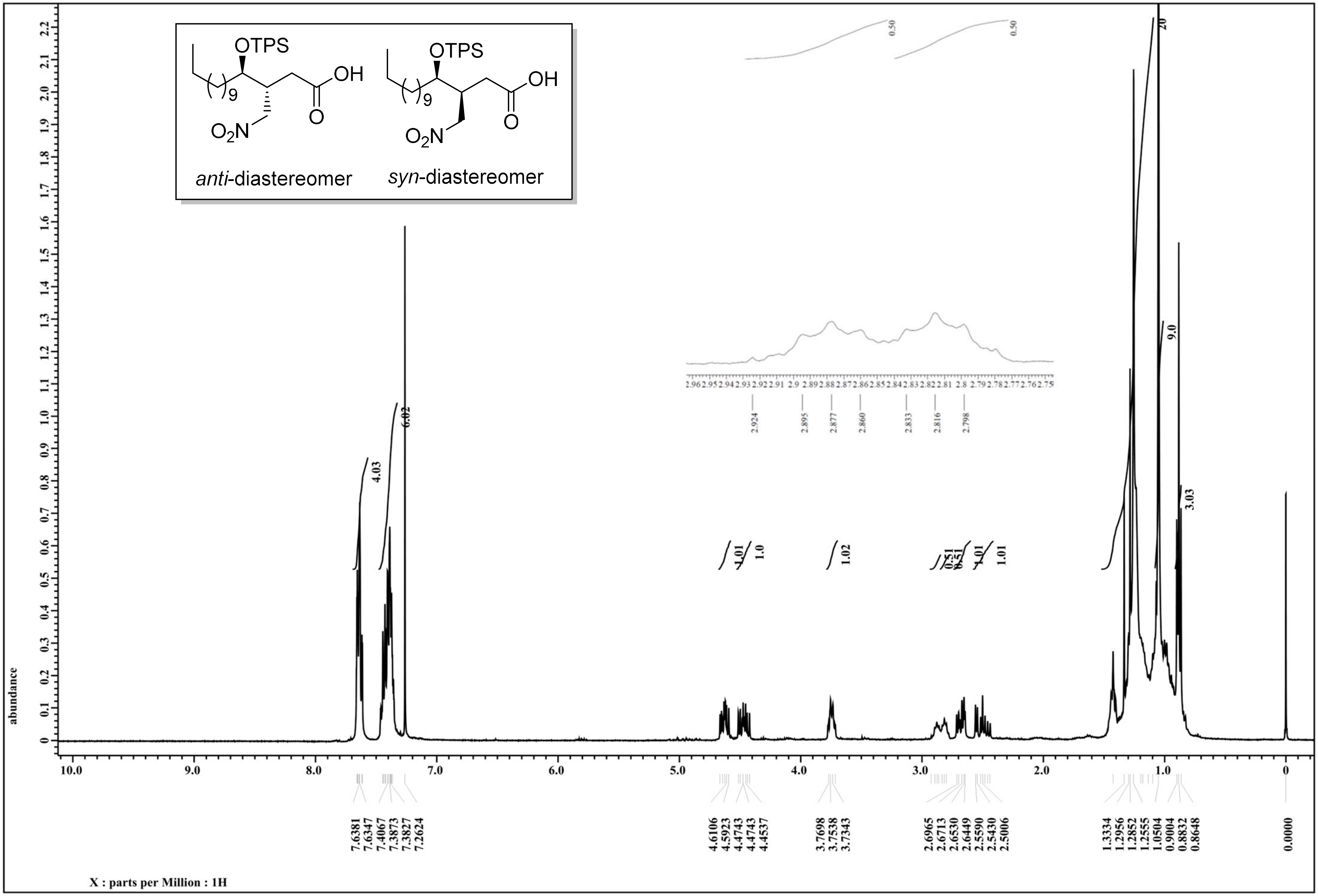

^{13}C NMR (100 MHz, $CDCl_3$/TMS)

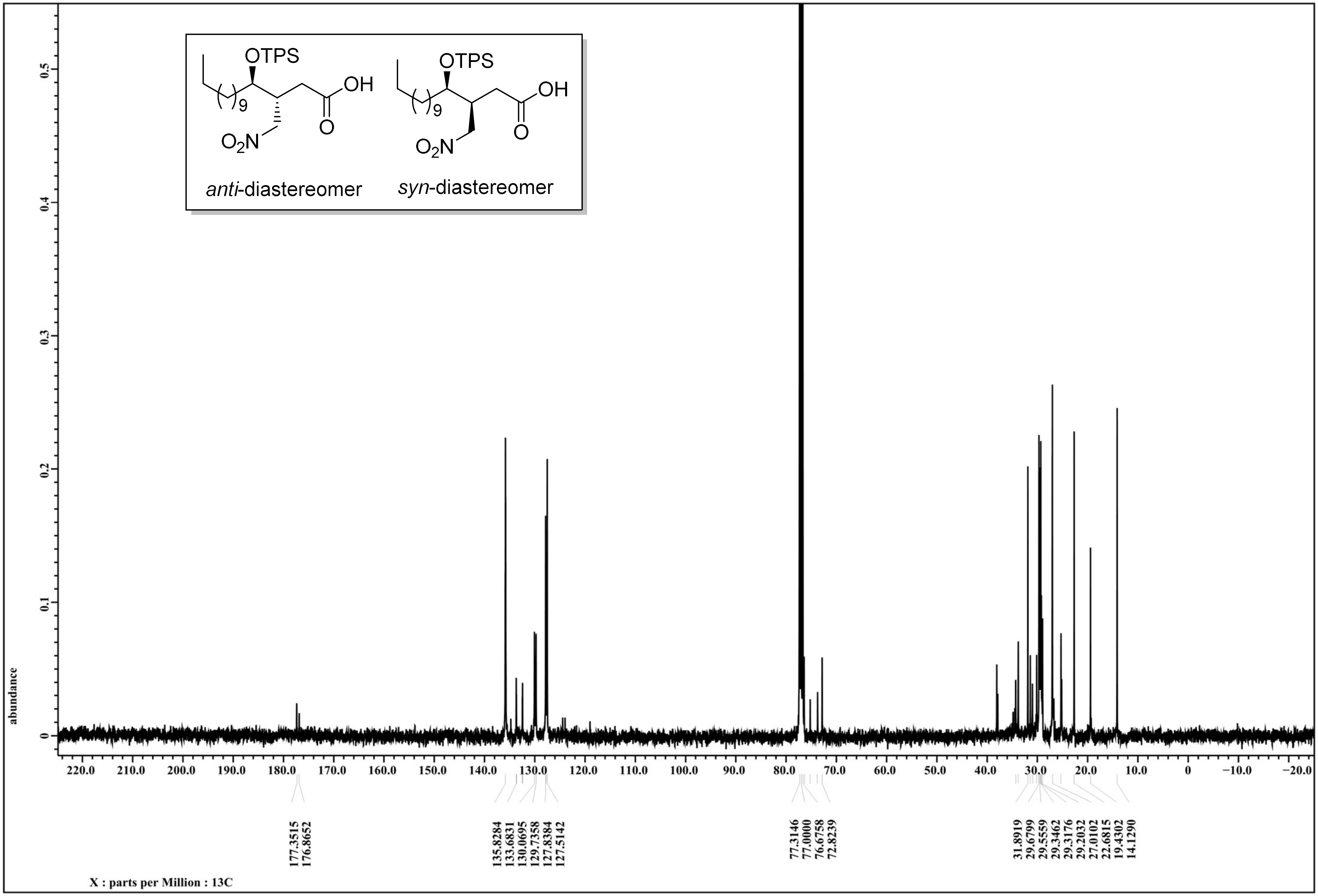

^{1}H NMR (400 MHz, $CDCl_3$/TMS)

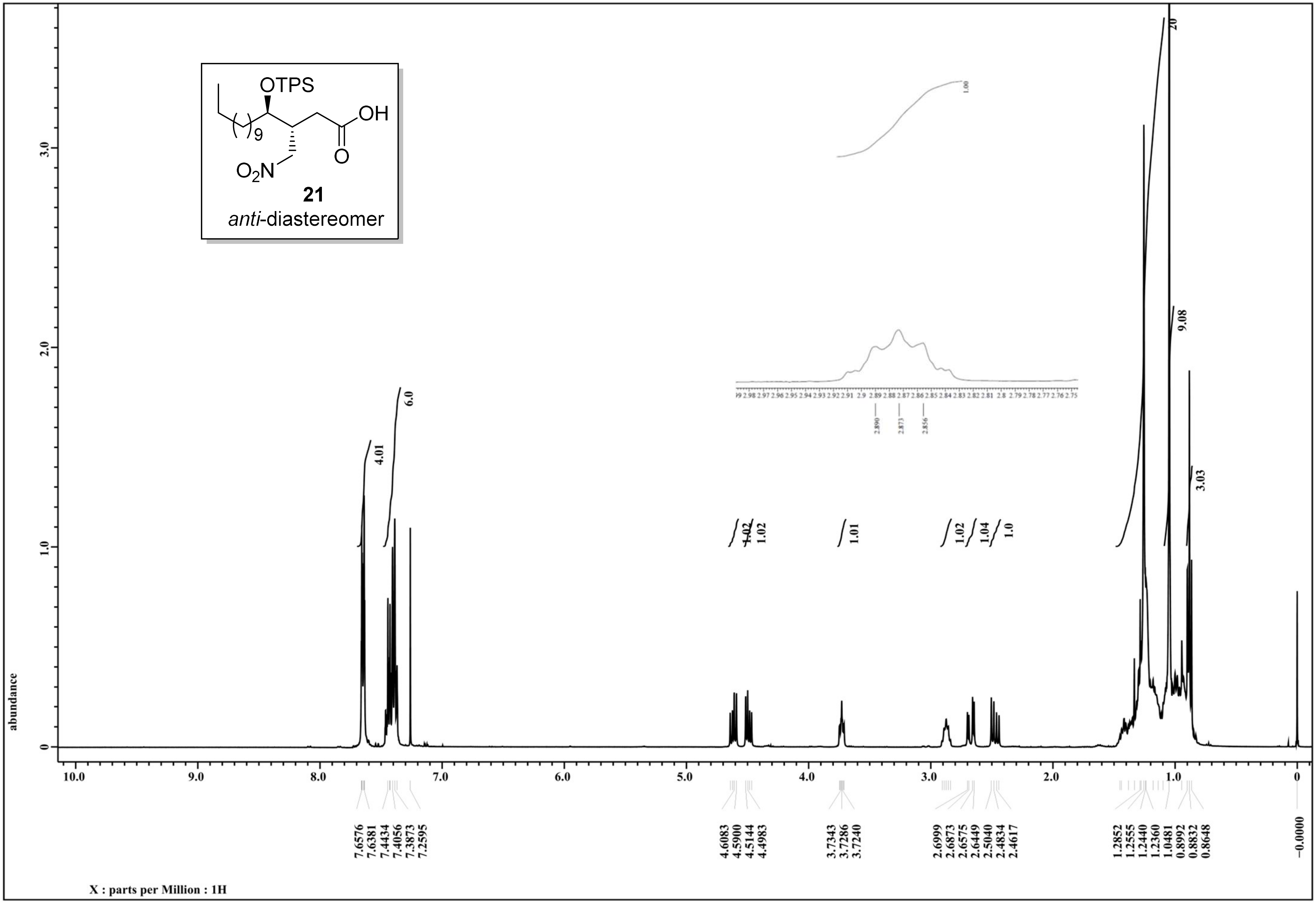

^{13}C NMR (100 MHz, $CDCl_3$/TMS)

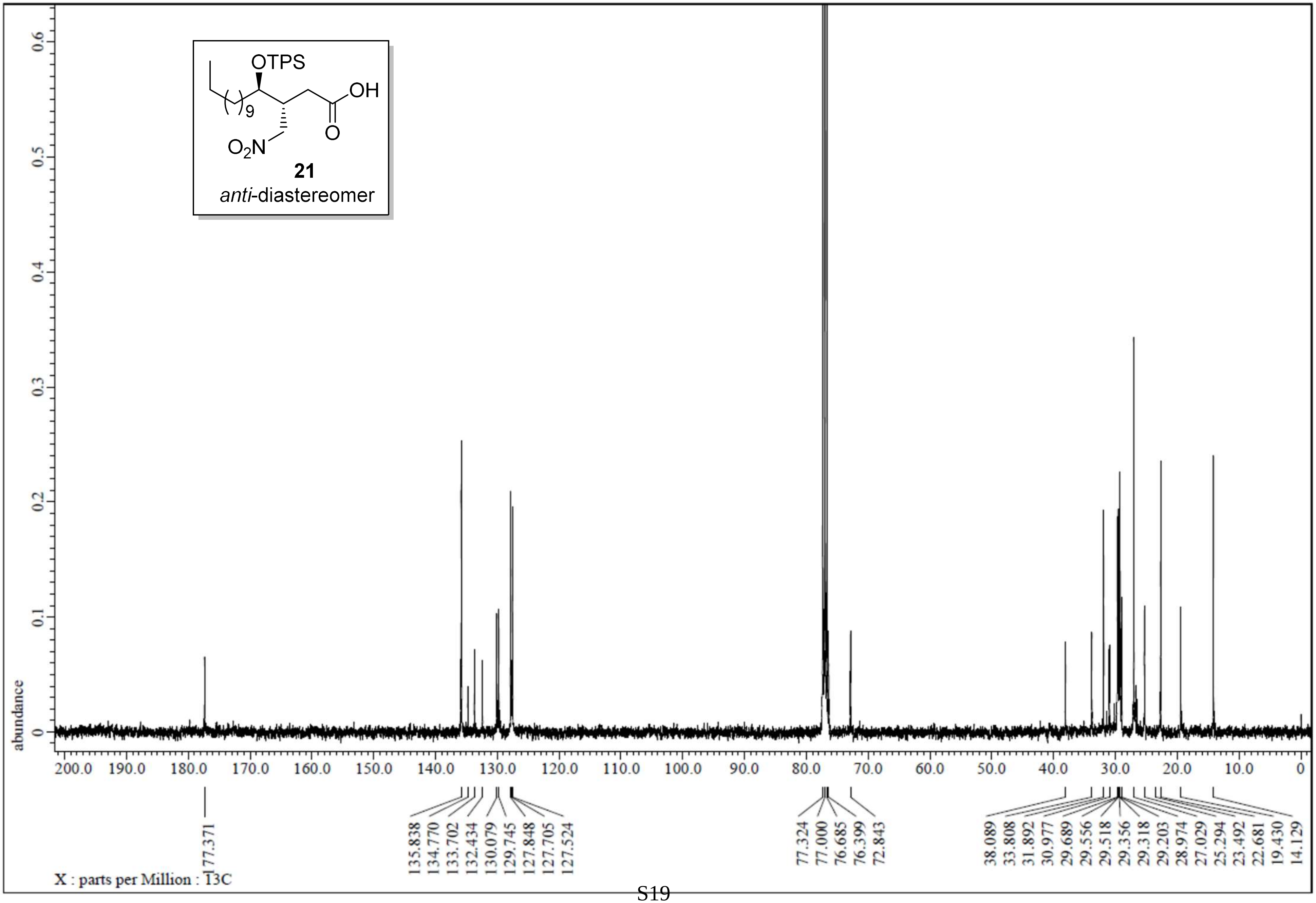

^{1}H NMR (400 MHz, $CDCl_3$/TMS)

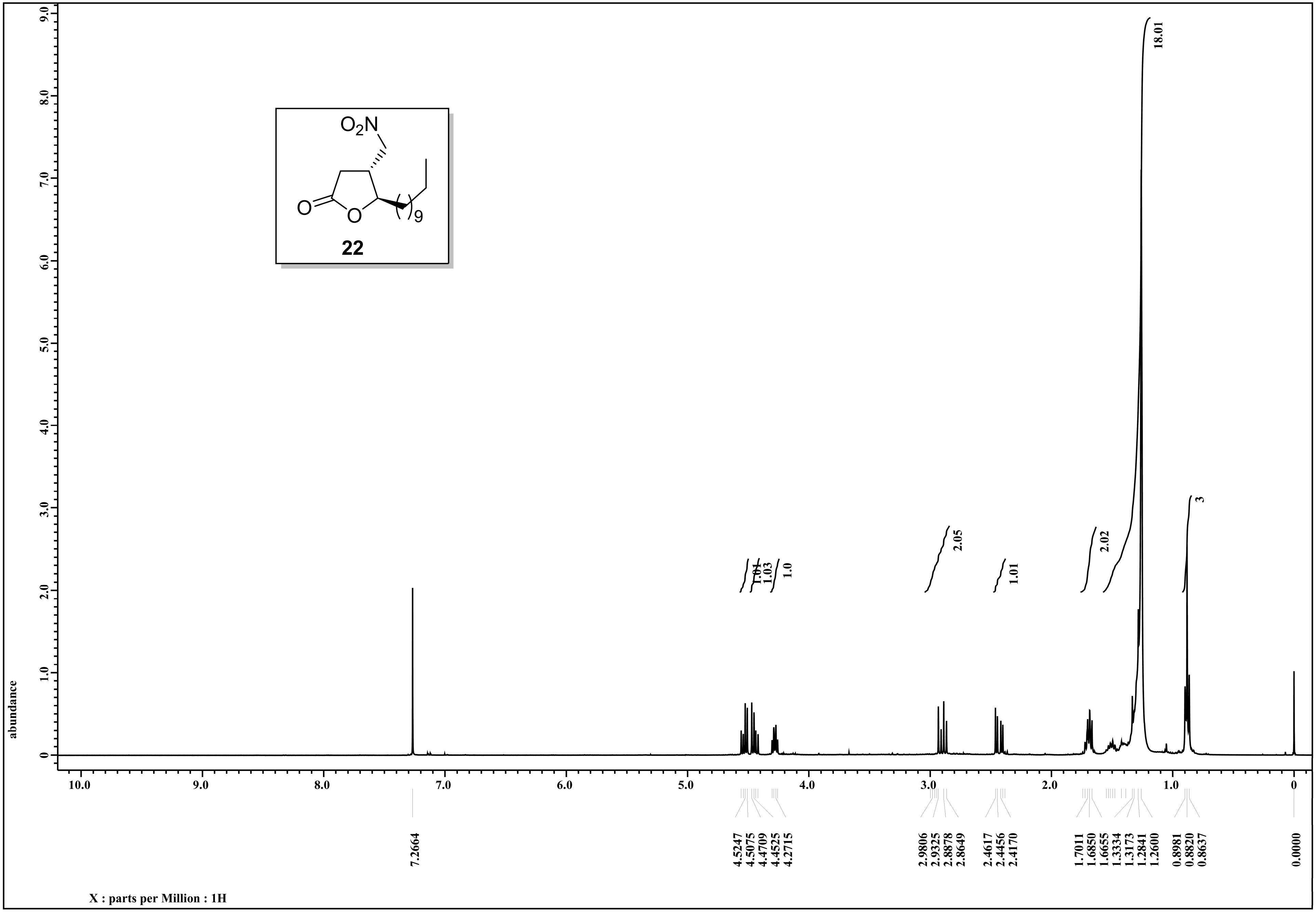

^{13}C NMR (100 MHz, $CDCl_3$/TMS)

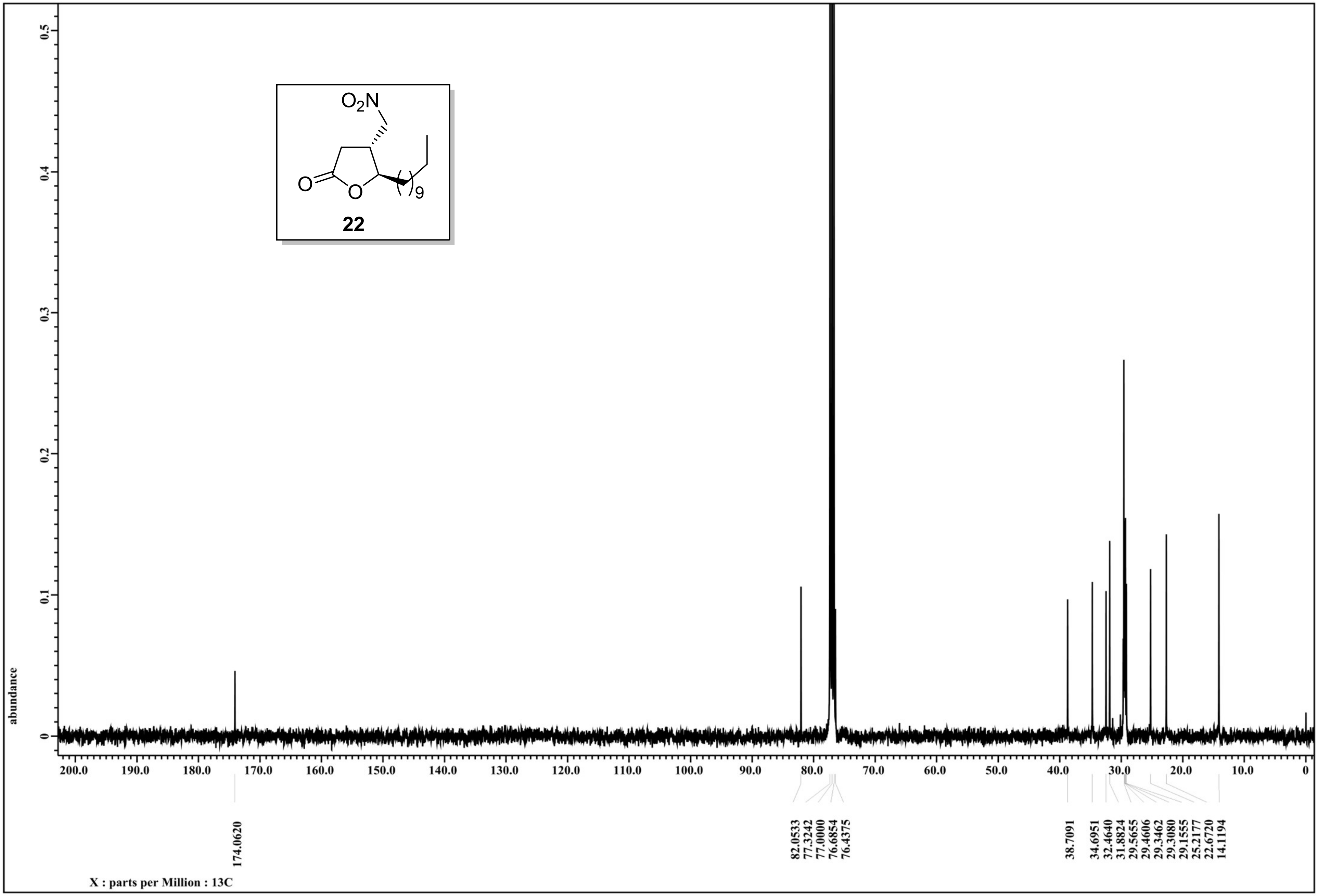

^1H NMR (400 MHz, $CDCl_3$/TMS)

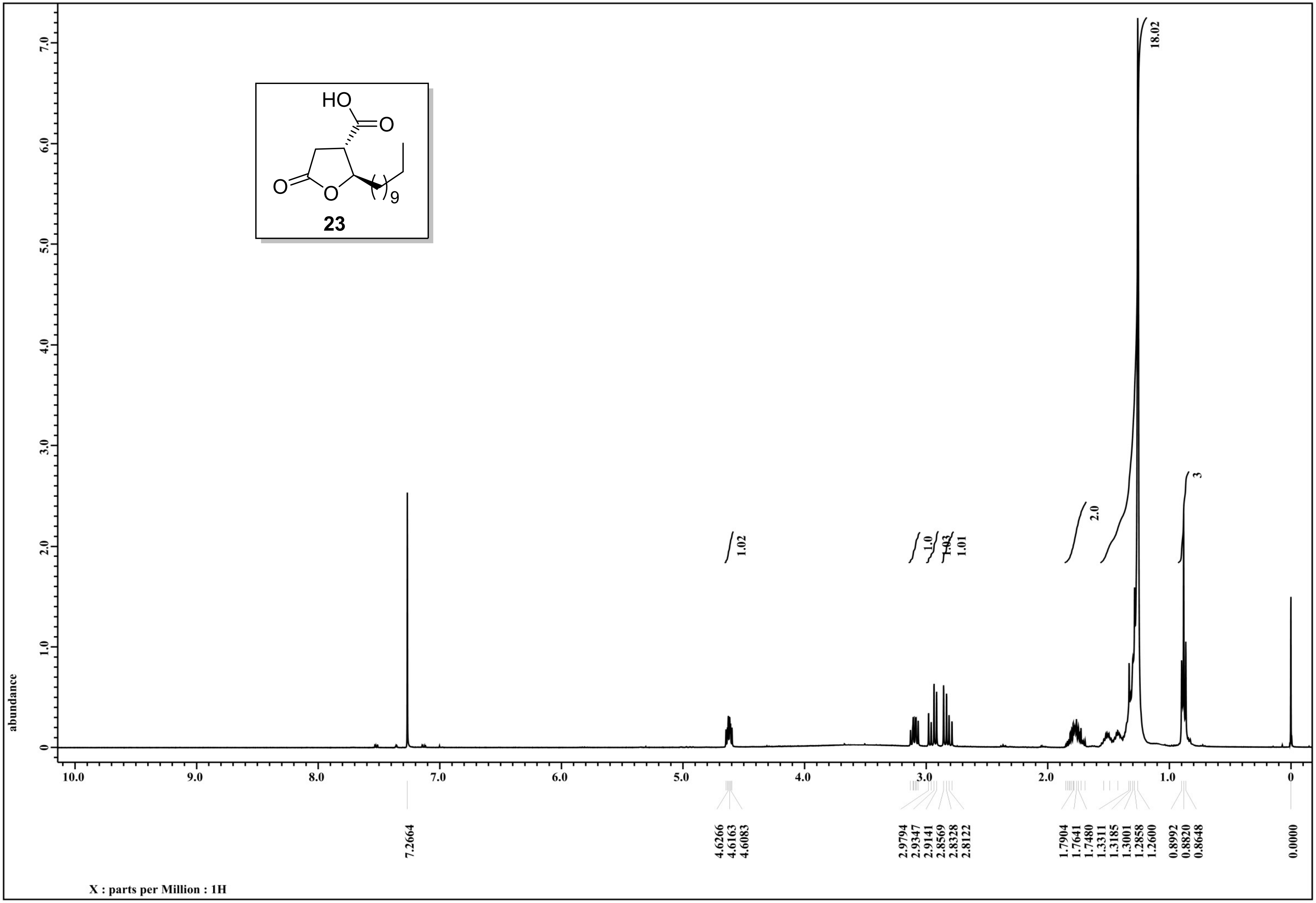

^{13}C NMR (100 MHz, $CDCl_3$/TMS)

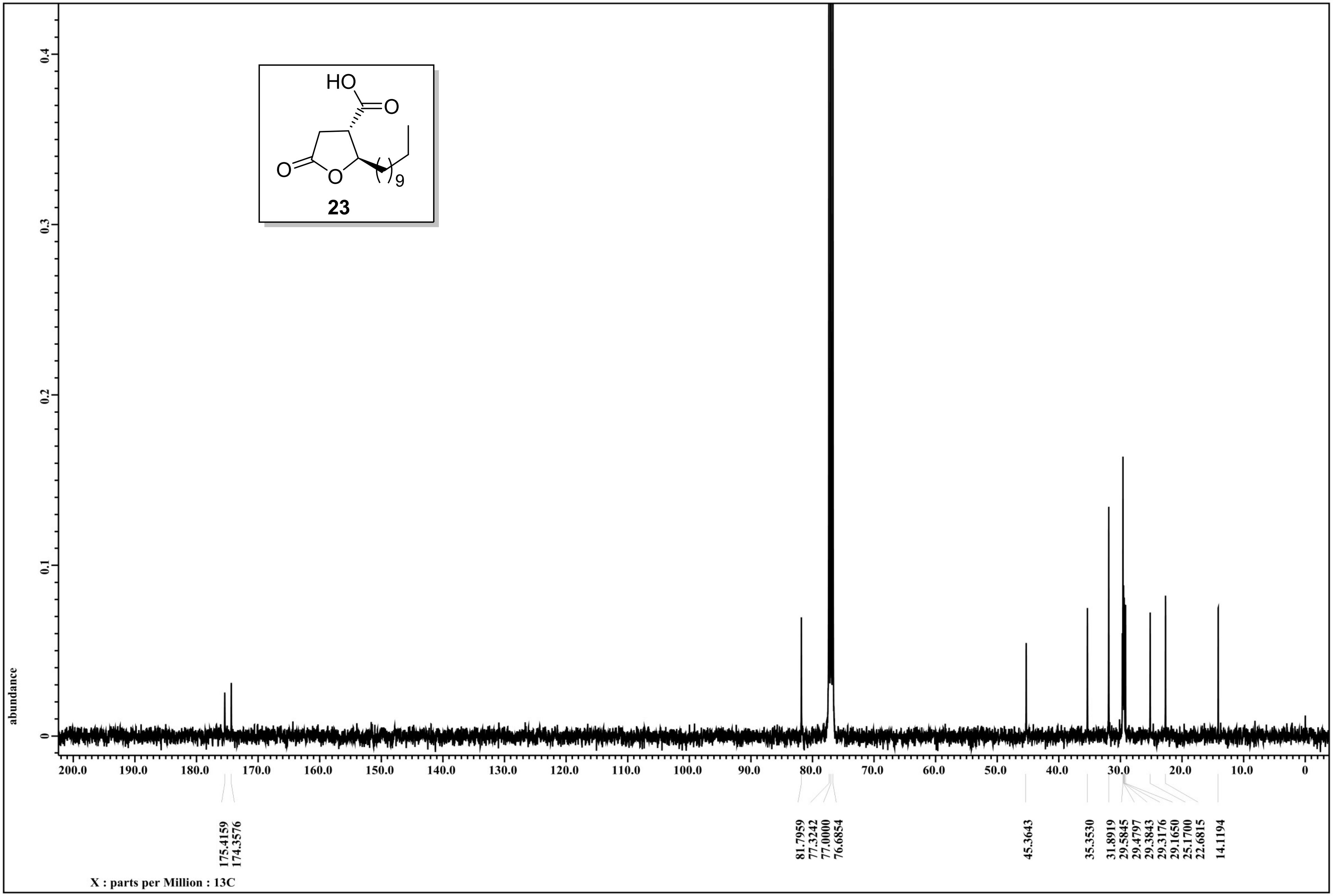

^{1}H NMR (400 MHz, CDCl/TMS)

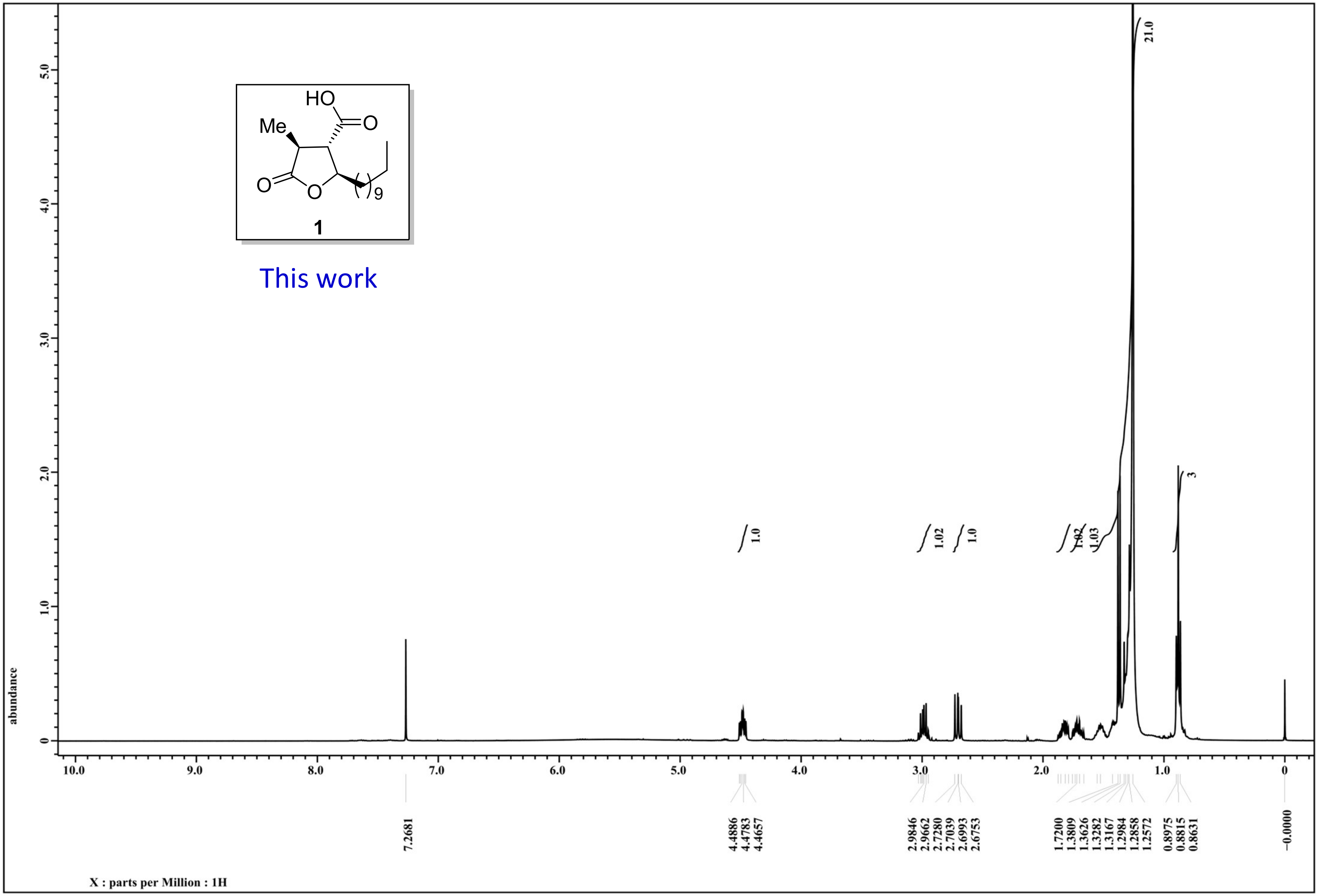

^{13}C NMR (100 MHz, CDCl3/TMS)

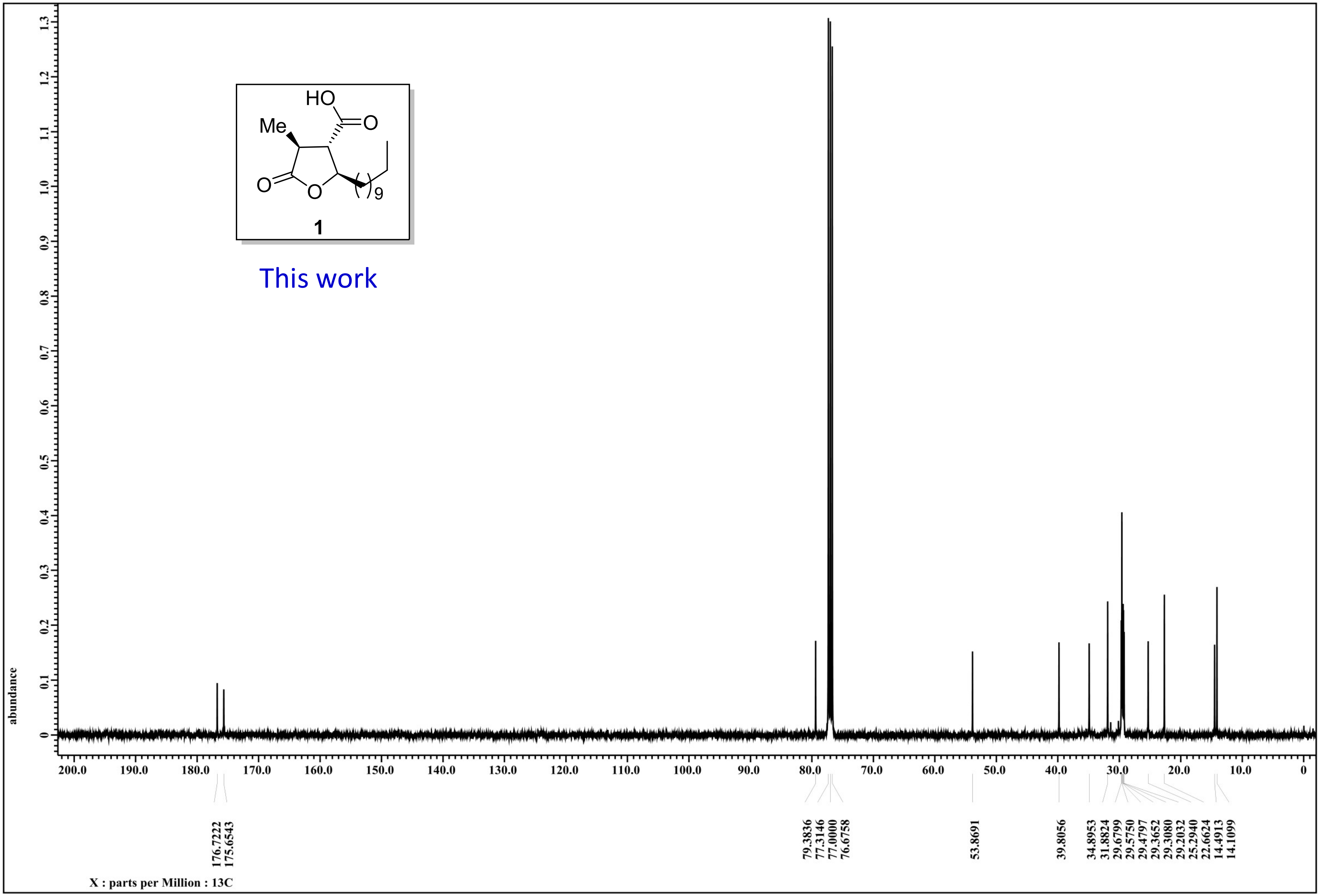

^{1}H NMR (400 MHz, $CDCl_3$) and ^{13}C NMR (100 MHz, $CDCl_3$) of **1**

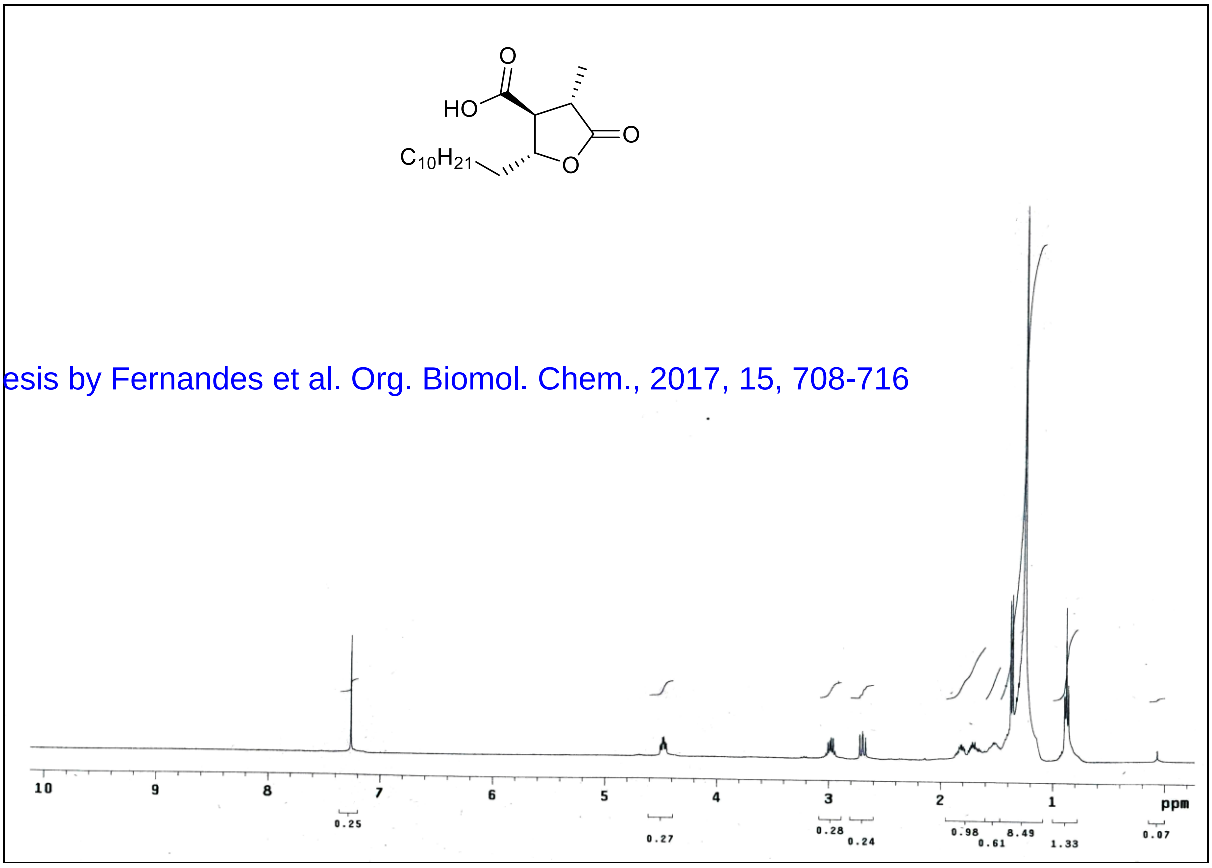

Synthesis by Fernandes et al. Org. Biomol. Chem., 2017, 15, 708-716

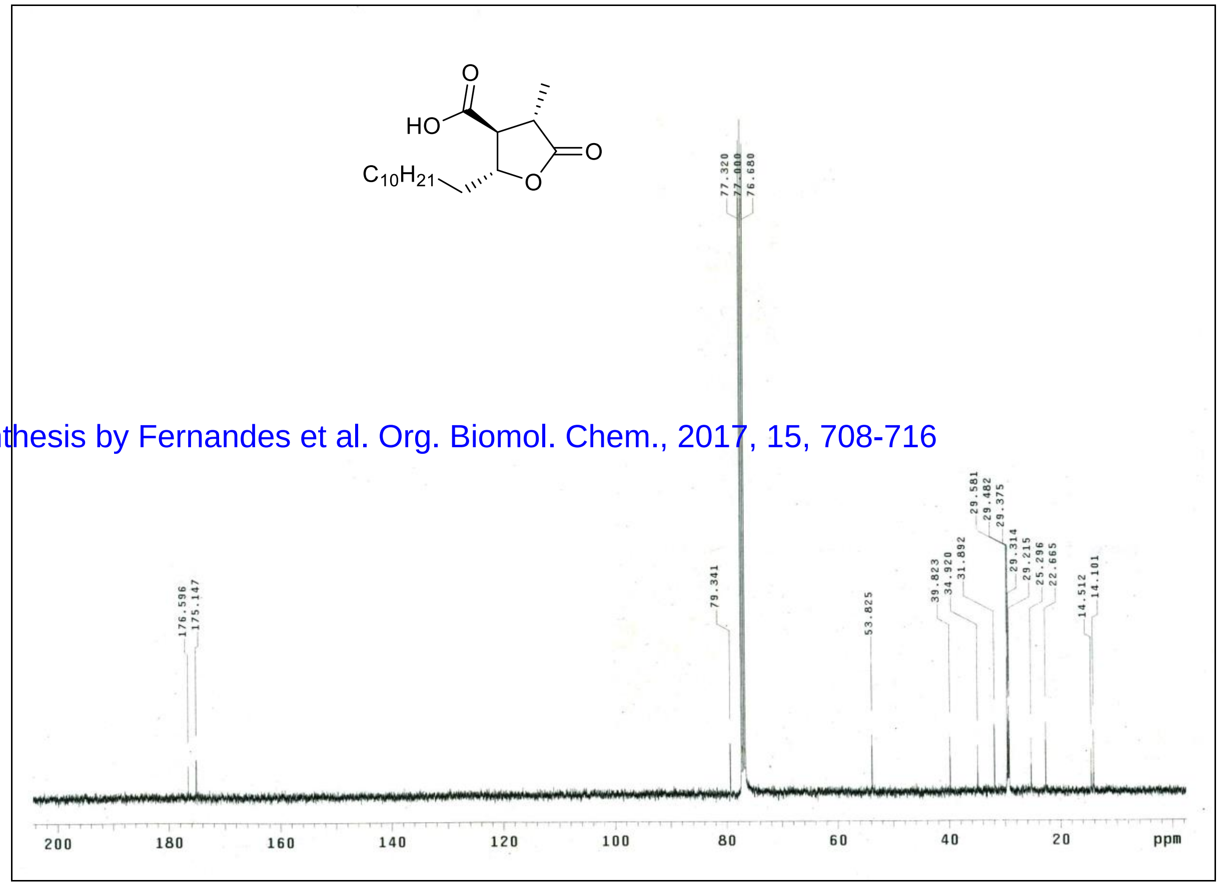

Synthesis by Fernandes et al. Org. Biomol. Chem., 2017, 15, 708-716
